# Multiscale 3D phenotyping of human cerebral organoids

**DOI:** 10.1038/s41598-020-78130-7

**Published:** 2020-12-08

**Authors:** Alexandre Albanese, Justin M. Swaney, Dae Hee Yun, Nicholas B. Evans, Jenna M. Antonucci, Silvia Velasco, Chang Ho Sohn, Paola Arlotta, Lee Gehrke, Kwanghun Chung

**Affiliations:** 1 Institute for Medical Engineering and Science, MIT, Cambridge, MA USA; 2 Picower Institute for Learning and Memory, MIT, Cambridge, MA USA; 3 Department of Chemical Engineering, MIT, Cambridge, MA USA; 4 Department of Brain and Cognitive Sciences, MIT, Cambridge, MA USA; 5 Department of Stem Cell and Regenerative Biology, Harvard University, Cambridge, MA USA; 6 Stanley Center for Psychiatric Research, Broad Institute of MIT and Harvard, Cambridge, MA USA; 7 Department of Microbiology and Immunobiology, Harvard Medical School, Boston, MA 02115 USA; 8 Harvard–MIT Program in Health Sciences and Technology, Cambridge, MA 02139 USA; 9 Center for Nanomedicine, Institute for Basic Science (IBS), Seoul, Republic of Korea; 10 Yonsei-IBS Institute, Yonsei University, Seoul, Republic of Korea

**Keywords:** Biological techniques, Biotechnology

## Abstract

Brain organoids grown from human pluripotent stem cells self-organize into cytoarchitectures resembling the developing human brain. These three-dimensional models offer an unprecedented opportunity to study human brain development and dysfunction. Characterization currently sacrifices spatial information for single-cell or histological analysis leaving whole-tissue analysis mostly unexplored. Here, we present the SCOUT pipeline for automated multiscale comparative analysis of intact cerebral organoids. Our integrated technology platform can rapidly clear, label, and image intact organoids. Algorithmic- and convolutional neural network-based image analysis extract hundreds of features characterizing molecular, cellular, spatial, cytoarchitectural, and organoid-wide properties from fluorescence microscopy datasets. Comprehensive analysis of 46 intact organoids and ~ 100 million cells reveals quantitative multiscale “phenotypes" for organoid development, culture protocols and Zika virus infection. SCOUT provides a much-needed framework for comparative analysis of emerging 3D in vitro models using fluorescence microscopy.

## Introduction

Embryonic and inducible pluripotent stem cells can differentiate into virtually any cell type from the body. Recently, organoid model systems have emerged from the optimization of three-dimensional differentiation protocols that drive the self-organization of cells into cytoarchitectures resembling tissue subregions. Cerebral organoids^1–3^ contain a mixture of cell types, intercellular interactions, and microenvironments that mimic early neurodevelopment from neuroepithelium formation to the assembly of rudimentary networks^2,4–7^. Each organoid contains dozens of ventricles lined with radial glia/progenitors that differentiate into cortical neurons and mature glia^2,8,9^. Organoids recapitulate genetic and epigenetic features of prenatal human brain development^5,10–12^ and have been used to identify the pathology of microcephaly^2,13^, lissencephaly^14,15^, Rett syndrome^16^, autism^17^, and Zika virus infection^9,18,19^ at the cellular scale.

Currently, organoid analysis relies predominantly on single-cell transcriptome analysis and 2D histology. Forfeit of 3D spatial information leaves systems-level single-cell analysis largely unexplored in cerebral organoids. Although protocols for whole-organoid tissue clearing have emerged^20,21^, there remains a considerable challenge in the quantification and interpretation of 3D datasets. The main challenge comes from the organoids’ inconsistent cell-patterning and overall morphology. Cerebral organoids are notoriously variable both within and between batches^5,8^. The optimization of more directed differentiation protocols has reduced organoid heterogeneity^9,12,22^. Nevertheless, all organoids show independent development of “neuroepithelial units” (ventricles) each producing their own cells and morphogen gradients^23^. Unlike animal and human brains where stereotypic development can be confidently analyzed in 2D based on a well-established common coordinate system, the random configuration of ventricles and the lack of common coordinates in organoids make it difficult to interpret cell biology and patterning in sampled subregions without any knowledge of the broader spatial context. This heterogeneity precludes usage of a “tissue atlas” which is common in volumetric tissue analysis since it standardizes the comparison of multiple datasets by establishing consistent tissue subregions. Thus, new technologies enabling atlas-free 3D whole-organoid analysis are required to achieve quantitative comparative analyses between experimental conditions.

Here, we present a computational pipeline termed Single-cell and Cytoarchitecture analysis of Organoids using Unbiased Techniques (SCOUT) for rapid and multiscale phenotyping of intact cleared cerebral organoids. The automated pipeline enables quantitative analysis of single cells, their spatial context, radial organization and whole-organoid features. SCOUT advances organoid analysis by enabling: (1) the analysis of previously inaccessible features, (2) the detection of rare events and (3) elimination of histological sampling bias. SCOUT also provides a framework for comparative analysis of ex vivo biological models. Instead of resorting to a reference atlas, we used strategic markers to identify major subregions and then developed a high-dimensional analysis based on ~ 300 descriptors that capture the phenotypic landscape of each organoid at multiple scales.

## Results

### Whole-organoid processing

**Figure 1:**
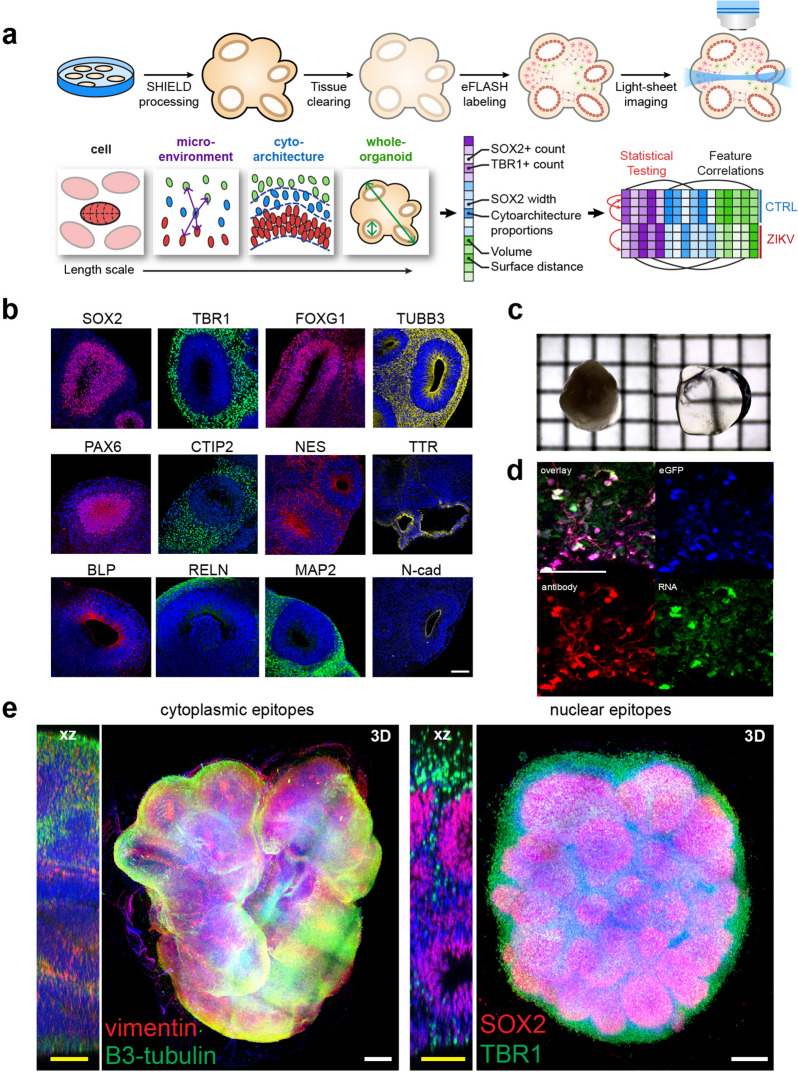
Pipeline for multiscale hyperdimensional analysis of organoids. (**a**) Scheme showing the pipeline where cerebral organoids are grown from stem cells, fixed in 4% PFA, then post-fixed with SHIELD poly-epoxide crosslinker. Organoids are then delipidated and labeled with antibodies using eFLASH for rapid uniform staining. An axially sweeping light-sheet microscope was used for rapid (15 min per organoid) imaging of cleared intact organoids at 0.65 × 0.65 × 2.0 µm voxel resolution. Quantitative analysis applies automated algorithms and convolutional neural networks to measure multiscale features. We applied this pipeline for unbiased high-dimensional phenotyping of different experimental models. (**b**) SHIELD is compatible with many common markers for cerebral organoids. Data show antibody staining of SHIELD-cleared day 45 organoids using antibodies listed in Supplementary Table 1. (**c**) After delipidation and immersion in refractive index matching solution (dPROTOS), organoids are optically transparent and can be imaged with standard confocal microscopy. Grid = 1 mm. (**d**) SHIELD preserves endogenous fluorescence, mRNA and protein epitopes. Images show an organoid section containing GFP-labeled cells co-stained with anti-GFP antibody and a GFP mRNA FISH probe (**e**) A 3D render of day 35 cerebral organoid stained using Syto16 (nuclear dye, blue), β3-tubulin (neuronal marker, green) and vimentin (glial marker, red) or Syto16 (nuclear dye, blue); TBR1 (postmitotic neuron, green); SOX2 (progenitor cells, red). Images show a 3D render and a 2D view of the XZ plane to show whole-tissue labeling. Scale bars (yellow, 100 µm; white, 200 µm).

We prepared cerebral organoids using an undirected protocol^24^ modified to include (1) dual SMAD inhibition during the first 3 days of neuroepithelium induction to increase neural conversion, and (2) BDNF addition after day 40 of culture to drive neuronal maturation^5,16,24,25^. Whole-tissue staining and imaging (Fig. 1a) was achieved by adaptation of the SHIELD protocol where poly-epoxide crosslinker preserves the tissue and its biomolecules during delipidation^26^. Epoxide groups in the polyglycerol 3-polyglycidyl ether molecule react with amines in the tissue to form stabilizing inter-and intra-molecular covalent bonds. SHIELD processing did not affect antibody staining of common cerebral markers such as BLP, FOXG1, SOX2, PAX6, N-Cadherin, Nestin, Reelin, TBR1, CTIP2, TTR, and MAP2 (Fig. 1b, Supplementary Fig. 1a, Supplementary Table 1). After immersion in PROTOS-based optical clearing solution^26^, cerebral organoids were optically transparent (Fig. 1c). SHIELD-cleared organoid tissue sections confirm the preservation of eGFP endogenous fluorescence, epitopes, and mRNA (Fig. 1d). For whole-organoid staining, we employed eFLASH technology^27–29^ to achieve simultaneous staining of 8–10 organoids in 1–2 days using a small amount of antibody (see “Methods” section). We achieved whole-organoid staining with various markers including SOX2, TBR1, MAP2, β3-tubulin, and Vimentin (Fig. 1e, Supplementary Fig. 1b, Video S1). Axially sweeping light-sheet fluorescent microscopy (LSFM) enabled three-channel imaging of intact organoids at single-cell resolution (0.65 × 0.65 × 2.00 µm voxel size) within approximately 15 min per organoid, generating approximately 150 GB of data per organoid (see “Methods” section).

### SCOUT analysis of single cells

**Figure 2:**
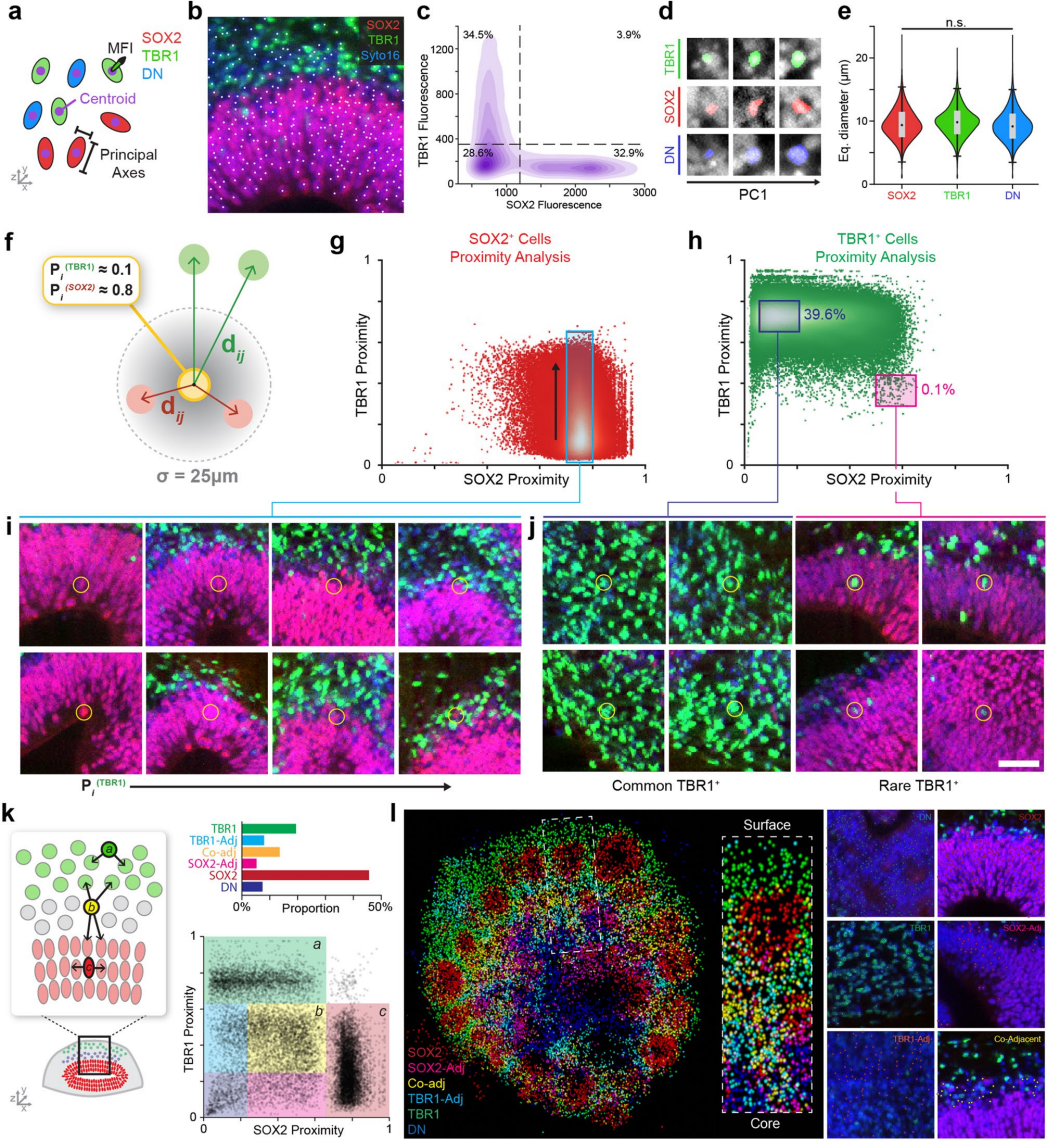
Single-cell detection and analysis. (**a**) Scheme of single-cell morphological characterization. (**b**) Demonstration of automated nuclei detection in 3D datasets. Syto16 in blue, SOX2 in red, TBR1 in green. Scale bar, 100 µm. (**c**) Expression of TBR1 and SOX2 in individual nuclei. Density plot shows gating parameters and population frequency. (**d**) Representative images of segmented nuclei for each cell type showing a range of morphological features sorted by principal component analysis. (**e**) Morphological analysis of individual nuclei shows a consistent equivalent diameter ~ 10 µm for all cell types. (**f**) Detailed scheme of the proximity analysis, which quantifies the distance of each cell’s nearest SOX2 and TBR1 cells using a 25 µm radius to normalize proximities. The proximity score between 0 (distant) and 1 (adjacent) reflects the cell’s spatial context as a function of progenitors and mature neurons. (**g**) Proximity analysis of SOX2 cells shows high proximity to other SOX2 cells. (**h**) Proximity analysis of TBR1 cells shows a high proximity to other TBR1 cells and a rare population of cells with a high SOX2 proximity. (**i**) Images showing the change in SOX2 cell position within the VZ as they increase their proximity score to TBR1 cells. Low proximity to TBR1 (left) reflects cells lining the ventricle whereas higher proximity shows cells at the edge of the ventricular zone (right). (**j**) Representative replicate images of TBR1 cells found in the major and rare populations of the proximity analysis. Scale bar = 50 µm. (**k**) Scheme of spatial context analysis showing the proximity of single cells to the nearest SOX2 and TBR1 cells. Bar graph shows the results of spatial context analysis shown below with “proximity score gates” to define six distinct populations. Gates captured > 99% of all cells. (**l**) Middle optical section of a day 35 organoid dataset showing detected cells colored according to their spatial context subcategorization. Inset shows zoomed view of dotted rectangle region. Right subpanels show instances of six different subpopulations identified by SCOUT.

After generating high-resolution 3D images, we sought to develop an analytical pipeline capable of converting our datasets into interpretable features. The goal was to capture each organoid’s “phenotype” based on features at multiple biological scales. For automated multiscale analysis of the 3D organoid datasets, we developed the SCOUT pipeline. All code for SCOUT and reproduction of our results are openly available on Github (https://github.com/chunglabmit/scout) or can be installed as a self-contained package using Docker (https://hub.docker.com/r/chunglabmit/scout). The basic principles of our analyses can be adapted and modified to suit other combinations of fluorescent markers, reporters, and highly-multiplexed molecular imaging modalities^29–33^. In this initial study, we deliberately selected dyes and antibodies that labeled nuclei to simplify cell segmentation and ensure accurate marker expression analysis. We used Syto16, a nucleic acid counterstain, anti-SOX2 to label radial glial progenitors and anti-TBR1 to label the early post-mitotic neurons (Fig. 2a,b). We chose these markers to delineate early organoids’ major subregions in the absence of a reference atlas. We applied these markers to quantify the frequency, position and organization of progenitors and early neurons to establish the ground work for image analysis methodologies.

The first step in 3D image analysis was the detection and segmentation of single cells. The high density of cells inside cerebral organoids caused unacceptable failure rates using conventional 3D “blob detection” algorithms. Instead, we applied an alternative approach using curvature-based seeded watershed on nuclear dye images^34^ and achieved an accuracy of 90% for nuclear detection (Supplementary Fig. 2). Detection was significantly better than other blob detector algorithms especially in regions of high cell density. Next, we co-localized segmented nuclei with SOX2 and TBR1 fluorescence for molecular phenotyping of individual cells (Fig. 2c). Since the expression of these two markers is mutually exclusive, we established specific fluorescence thresholds to identify: SOX2^+^ progenitors, TBR1^+^ neurons, and double negative (DN) cells. DN cells may include intermediate progenitor cells, TBR1-negative neurons, and apoptotic cells depending on the developmental stage of organoids and experimental conditions used in this study. Morphological analysis of individual nuclei shows a ~ 10 µm average diameter for all three cell types, although we detect a range of different volumes (Fig. 2d,e).

Single-cell datasets provided spatial coordinates and marker expression, which offered new opportunities to interrogate the spatial context of individual cells. We developed a “spatial context” descriptor for every cell by computing a positional proximity score (P_i_) based on each cell’s distance to its nearest SOX2^+^ and TBR1^+^ cells (see “Methods” section). In essence, this score quantified each cell’s proximity to SOX2-rich ventricular zones (VZ) and neuron-rich TBR1 regions (Fig. 2f,k). Most SOX2^+^ cells showed high proximity (P_i_^SOX2^ > 0.65) to other SOX2^+^ cells (Fig. 2g,i). We observed the same phenomenon with TBR1^+^ cells (Fig. 2h,j). These findings highlight the proximity score’s ability to identify SOX2^+^ and TBR1^+^ regions in highly patterned tissues. Interestingly, we detected a range of proximities to TBR1^+^ cells among SOX2^+^ cells (P_i_^SOX2^ = 0.7–0.8). Proximity to TBR1 distinguished SOX2 cells bordering the ventricle’s lumen (P_i_^TBR1^ < 0.1), in the middle of the VZ (P_i_^TBR1^ = 0.2–0.4) or adjacent to TBR1 cells (P_i_^TBR1^ = 0.5) (Fig. 2i). Analysis of the TBR1^+^ cells, revealed a rare subset with a high SOX2 proximity (P_i_^SOX2^ = 0.6) corresponding to TBR1^+^ cells inside the SOX2^+^-rich VZ (Fig. 2j). These rare cells may represent newly generated neurons migrating out of the VZ or minor defects in organogenesis.

Analysis of DN cells reveals that cells in the featureless core of the organoid (away from the ventricles) showed the lowest SOX2 and TBR1 proximity scores (P_i_^SOX2^, P_i_^TBR1^ < 0.15) (Fig. 2k). DN cells also segregated into three additional subpopulations: SOX2-adjacent cells in the VZ, TBR1-adjacent cells among the neurons and SOX2/TBR1 co-adjacent cells. The location of these DN subpopulations helps to infer their identity given that TBR1-adjacent DN cells are most likely postmitotic TBR1^-^ neurons and a SOX2-adjacent DN cells may represent intermediate progenitors*.* Our proximity analysis demonstrates a novel strategy for enhanced cell phenotyping by combining molecular markers and spatial context for automated high-dimensional characterization in whole organoids.

### SCOUT analysis of regional architectures

**Figure 3:**
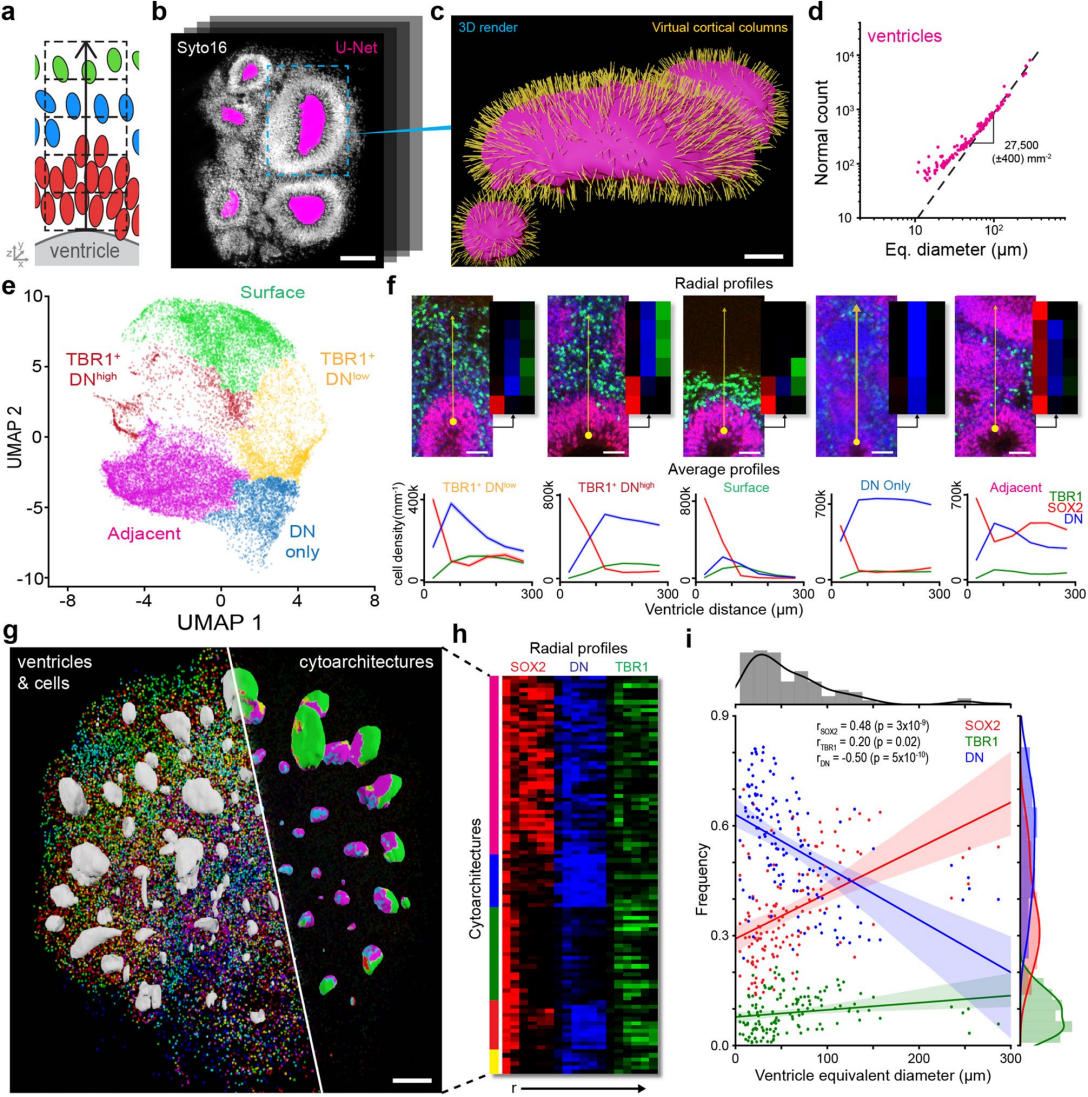
SCOUT analysis of regional architectures. (**a**) Scheme of automated cytoarchitecture analysis. We quantified radial organization of cell populations around ventricles using “virtual cortical columns” 50 µm in diameter and 300 µm high, perpendicular to the ventricle surface. (**b**) Demonstration of automated ventricle segmentation using U-Net convolutional neural network. Representative optical section of a volumetric dataset with detected ventricles in magenta. (**c**) A 3D render of ventricle highlighted in panel B with normals used to orient virtual cortical columns shown in yellow. (**d**) Graph showing that the total number of normals per ventricle depends on the ventricle’s surface area. (**e**) UMAP embedding of detected cytoarchitectures in a single organoid color-coded according to each cluster. (**f**) Representative image and average profile plot of individual cytoarchitecture clusters showing the radial distribution of SOX2 (red), double negatives (blue) and TBR1 (green) cells. Scale bar, 50 µm (**g**) 3D render of segmented cells and ventricles from a day 35 organoid. On the left side ventricles are white and six cell populations are colored according to the index in Fig. 2l: SOX2 in red, SOX2-adjacent in magenta, co-adjacent in yellow, TBR1-adjacent in cyan, TBR1 in green and core DN in blue. On the right, we mapped the detected cytoarchitectures on the surface of rendered ventricles using the colors in (**f**). Scale bar = 200 µm (**h**) Three-channel heat map from 100 random cytoarchitectures. Each row shows the number of cells detected in all six 50 µm increments moving away from the ventricle surface. Intensity of red, blue and green represent SOX2, DN and TBR1, respectively. (**i**) The frequency of SOX2, DN and TBR1 cells detected in a ventricle’s virtual cortical columns correlates with the ventricle equivalent diameter. Strongest correlation occurs for decreased DN and increased SOX2 in larger ventricles.

Next, we sought to characterize cell organization (cytoarchitectures) in the organoids (Fig. 3). In both the developing brain and cerebral organoids, cells organize around the ventricle as new cortical neurons migrate along radial glia fibers. In previous studies, SOX2^+^ and TBR1^+^ cell position relative to the ventricle enabled morphological analysis of radial patterning and delineation of cortical structures such as the ventricular zone^9,18^. The 3D analysis of radial cytoarchitectures requires the segmentation of disparate ventricle lumens in each organoid to establish the origin and direction of radial patterning. Thus, we adapted the architecture of a convolutional neural network, called U-net^35^, to detect SOX2-lined ventricle lumens based on manually segmented datasets (Supplementary Fig. 3, see “Methods” section). Neural network-based ventricle segmentation achieved a Dice coefficient of 97.2% and enabled basic morphological analysis (volume, axis ratio, etc.) of the three-dimensional ventricles (Fig. 3b).

Segmented ventricles surfaces were then used to establish the starting point of radial patterning. We quantified the radial organization of cell populations by generating “virtual cortical columns” perpendicular to the ventricle’s surface 50 µm in diameter and 300 µm long (Fig. 3a–c). Each column captures SOX2, TBR1, and DN cell counts in six equal subdivisions of the column. We generated thousands of columns uniformly distributed across the surface of all ventricles in a single organoid for comprehensive quantification of radial cytoarchitectures in the organoid. As expected, the number of columns generated per ventricle was proportional to its surface area (Fig. 3d). The next step was the generation of clusters to distinguish between cytoarchitectures. Unsupervised hierarchical clustering of the data after UMAP embedding (to reduce dimensionality^36^) revealed five distinct cytoarchitectures that we named TBR1^+^DN^low^, TBR1^+^DN^high^, Surface, DN only, and Adjacent based on their features (Fig. 3e,f). TBR1^+^DN^low^ and TBR1^+^DN^high^ possess a neuronal layer and only vary in the overall abundance of DN cells. “Surface” cytoarchitectures also contained a layer of TBR1^+^ neurons followed by a cell-free region where the virtual cortical columns projected into the empty space above the organoid’s surface. “Adjacent” cytoarchitectures possessed two SOX2 peaks due to the presence of another VZ less than 250 µm away. “DN only” cytoarchitectures consisted of mostly DN cells with scant SOX2 cells close to the ventricle.

Mapping cytoarchitectures onto the surface of the ventricles produced noticeable trends such as “DN only” appearing exclusively in small ventricles at the organoid’s featureless core and “Surface” appearing on large ventricles facing the tissue’s surface (Fig. 3g,h). To quantify potential correlations between cytoarchitectures and a ventricle’s properties, we quantified cell-type frequency for individual ventricles using cell data from their virtual columns (Fig. 3i). Analysis showed a significant correlation between ventricle size and SOX2 frequency due to a thicker VZ surrounding larger ventricles. We also detected a correlation between ventricle size and TBR1 cell frequency, suggesting that larger ventricles are farther along in cortical development. In summary, SCOUT quantified the frequency of cytoarchitectures within the organoid using nuclear epitopes and provided ventricle-specific feature extraction for downstream analysis.

### SCOUT whole-organoid analysis and correlation

**Figure 4:**
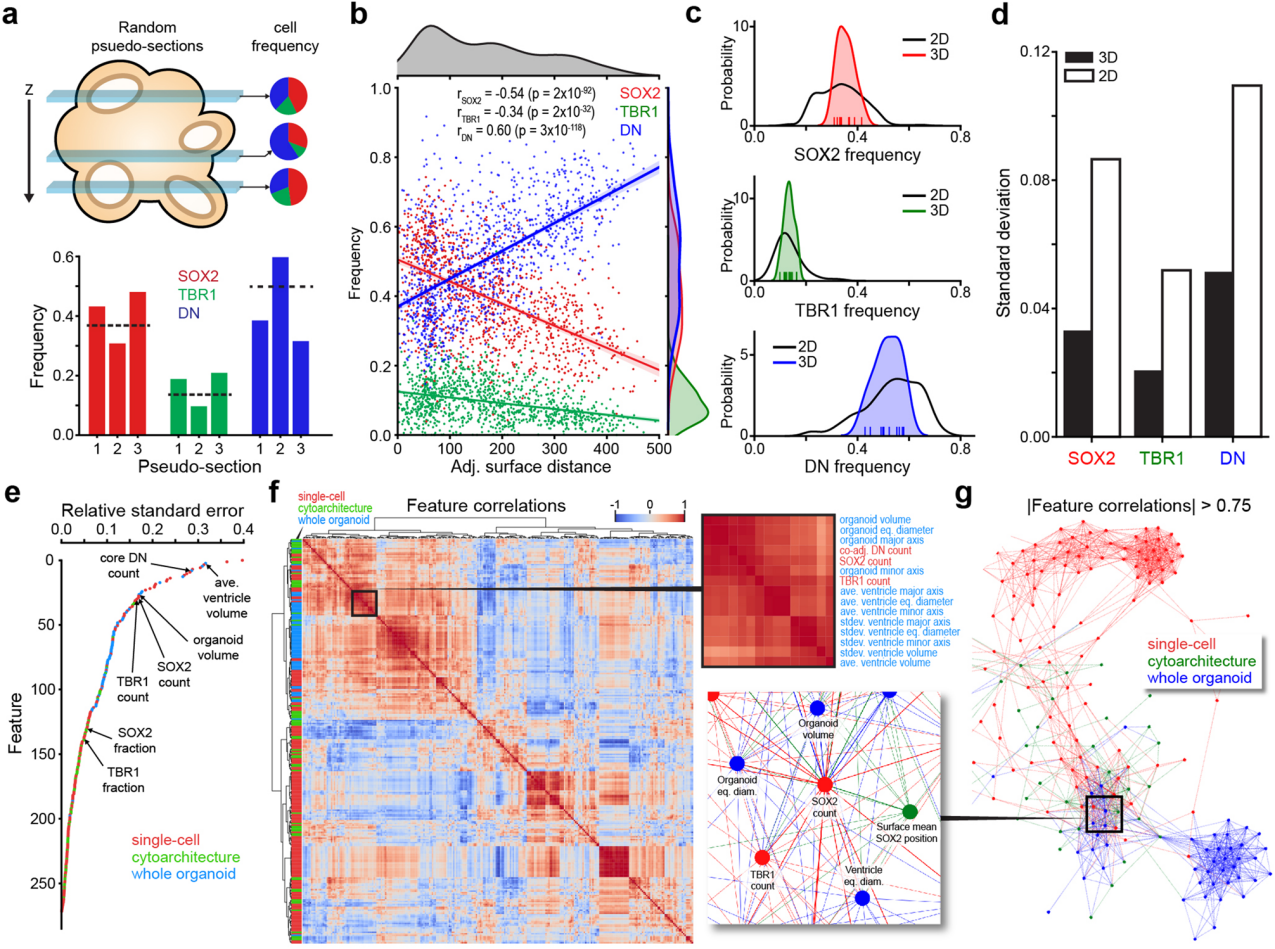
Whole-organoid analysis for unbiased quantitative studies. (**a**) Scheme (top) and analysis of three different 100 µm pseudo-sections from a 3D dataset. Pie charts (right) show the variable depth-dependent cell frequency for individual slices. Bottom shows the estimated cell frequency for each cell type with the actual whole-organoid frequency (dotted line). (**b**) Analysis of cell frequencies for 1187 segmented ventricles pooled from 12 day 35 organoids. Cell frequency for each ventricle was determined by combining the counts of cells detected in all “virtual cortical columns” used for cytoarchitecture analysis. (**c**) Comparison of pseudo-slice heterogeneity with biological inter-organoid variability. Each histogram shows the distribution of 10,000 pseudo-sections. 100 µm thick for 12 organoids (black) versus the distribution of whole-organoid frequency in different replicates (colored histogram, ticks show independent organoid values). (**d**) Comparison of cell frequency standard deviation for the pseudo-sections sampling variability (2D) versus whole-organoid biological variability (3D) in 12 organoids. (**e**) Comparison of the relative standard error for 276 multiscale features in 12 ‘day 35’ organoid replicates where each dot is colored based on to the scale of its analysis. (**f**) Heat map of Pearson’s correlation coefficient investigating the relationship between the 276 multiscale features and their variation in  ‘day 35’ organoid replicates. Right, shows the cropped region where we see a combination of single-cell (red) and whole-organoid (blue) features where r > 0.88. (**g**) Network of feature correlation when the absolute Pearson’s correlation coefficient is > 0.75. Cropped region shows the same multiscale correlation as the heat map in panel F. The SOX2 cell count and organoid volume were the most central nodes in this cluster, both having a degree of 30.

With our ability to measure the absolute frequency of different cell populations in individual organoids, we wanted to quantify the accuracy of conventional histological analysis based on two-dimensional tissue sections. We performed this experiment in silico by generating 100 µm-thick “pseudo-sections” from 3D datasets to determine how well they predicted whole-organoid cell frequency (Fig. 4a). The pseudo-sections incorrectly estimated the organoid’s cell type frequencies and generated significant variability due to each pseudo-section’s position within the organoid. Cell type frequency of each ventricle correlated with distance from the organoid surface producing a positional bias for the three cell populations (Fig. 4b). TBR1^+^ and SOX2^+^ frequencies were highest at the organoid surface then decreased with depth. Inversely, DN cell frequency increased in deeper ventricles and reached a maximum of 75% near the organoid’s core. These depth trend mirrored the correlation between ventricle size and the frequency of SOX2^+^ and TBR1^+^ cells (Fig. 3i) since larger ventricles were generally closer to the surface. When comparing the variability introduced by pseudo-section sampling to the biological heterogeneity of cell frequency in replicate organoids (n = 12), we observed a two- to three-fold increase in standard deviation for the pseudo-sections (Fig. 4c,d).

We further explored inter-organoid differences by combining our multiscale feature analysis into a set of 276 descriptors quantifying single cell, cytoarchitecture and whole-tissue features for each organoid (Supplementary Table 2). Comparative analysis of 12 organoids revealed unequal variance in their features. Relative standard error comparisons reveal that the most variable features included average ventricle volume, DN cell total count, and organoid volume (Fig. 4e). Interestingly, the frequencies of SOX2^+^ and TBR1^+^ cells were less variable than their total number. Given the high variability of certain features, we computed pairwise correlations between multiscale features to see if single-cell measurements could predict whole-tissue topography (Fig. 4f). Multiscale correlation occurred around SOX2 and TBR1 cell counts. Network analysis of pairwise correlations (r > 0.75) confirmed this multiscale correlation where “SOX2 count” and “organoid volume” were central nodes (degree of 30). Variation in SOX2 count correlated with ventricle size, TBR1 counts, co-adjacent DN counts and average SOX2 distance from the surface (Fig. 4g). These analyses demonstrate that SCOUT can reduce sub-sampling variance, quantify the previously inaccessible biological heterogeneity of replicates, and provide a framework correlating multiscale features.

### SCOUT analysis of organoid development

**Figure 5:**
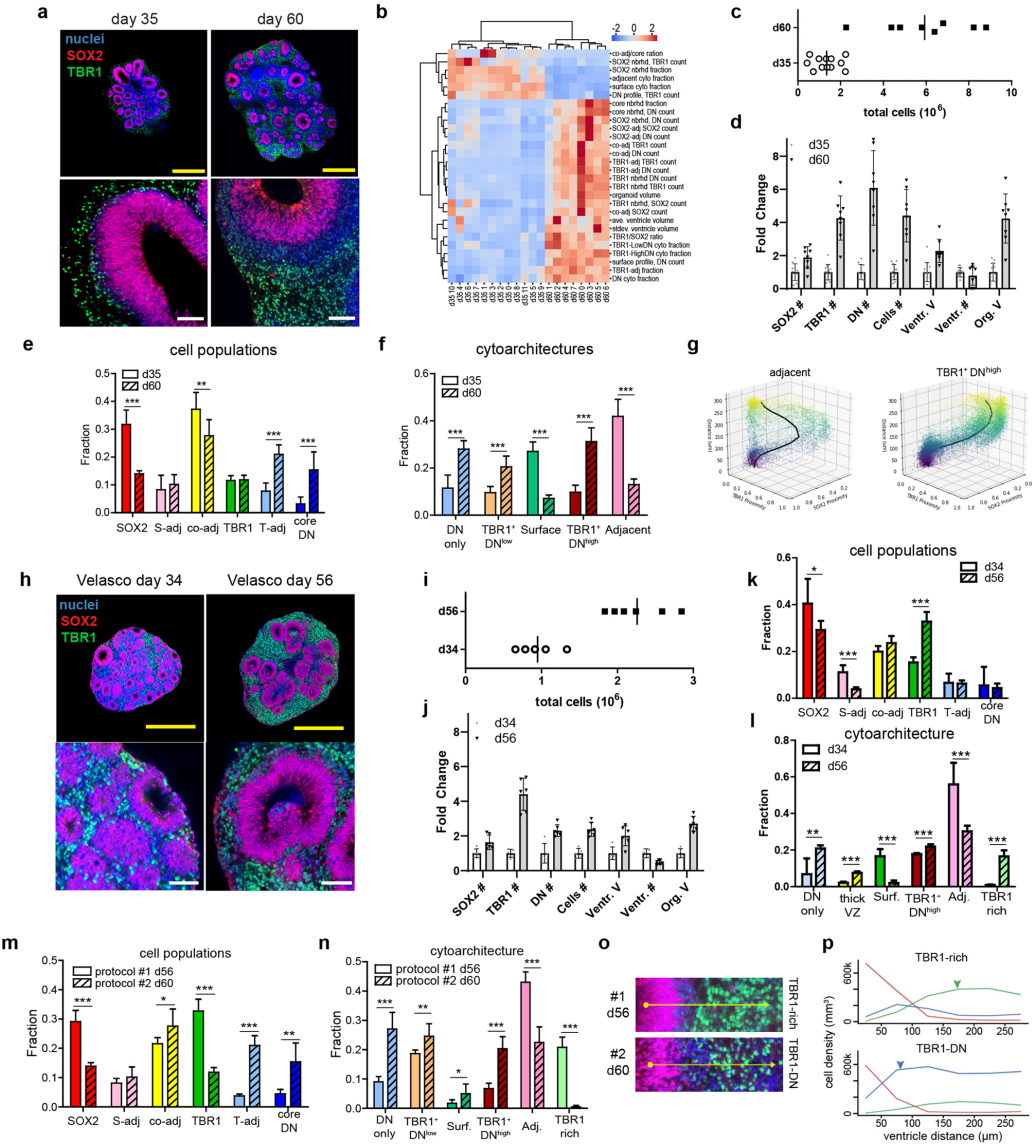
Hyper-dimensional analysis of multiscale changes during cerebral organoid development. (**a**) Representative image of day 35 and day 60 undirected organoids taken from volumetric datasets. Scale bars yellow = 1 mm and white = 100 µm (**b**) Heat map outlining major differences between day 35 (n = 12) versus day 60 (n = 8) organoids. (**c**) Dot plot showing total cell counts for all organoid replicates in each age group. (**d**) Fold-change in total cells, average ventricle volume, and total counts of cell populations. (**e**) Average frequency of cell subpopulations and (**f**) cytoarchitecture clusters in different age groups (**g**) Analysis of cell proximity to SOX2 and TBR1 in “adjacent” (left) and “TBR1, DN^high^” (right) as a function of the distance from the ventricle surface. (**h**) Representative image of Velasco organoids at day 34 (n = 5) and day 56 (n = 6) taken from volumetric datasets. Scale bars: yellow = 1 mm and white = 100 µm (**i**) Dot plot showing total cell counts of Velasco organoid replicates in each age group. (**j**) Fold-change in total cells, average ventricle volume, and total counts of antibody-labeled subsets in Velasco organoids. (**k**) Average frequency of cell subpopulations and (**l**) cytoarchitecture clusters in different age groups of Velasco organoids. (**m**) Comparative analysis of cell subsets in day 60 organoids from panel a (protocol #1) and d56 Velasco patterned organoids from panel h (protocol #2). (**n**) Comparison of cytoarchitecture clusters in protocols 1 and 2. (**o**) Representative image of cytoarchitecture most common in protocol 1 (TBR1-rich) and protocol 2 (TBR1^+^DN^high)^ organoids. Arrow is a virtual cortical column 300 µm in length. (**p**) Radial distribution of SOX2 (red), double negatives (blue) and TBR1 (green) cells in the cytoarchitectures shown in subpanel o. Arrows show the higher TBR1 frequency (green) and DN band between SOX2 and TBR1 (blue). [***p < 0.001, **p < 0.01, *p < 0.05].

With the development of a framework for comparative analysis, we used SCOUT to quantify development-related multiscale changes between day 35 and day 60 cerebral organoids (Fig. 5, Supplemental Fig. 4; Videos S1 and S2). We compared multiscale features between groups using independent two-tailed t-tests and detected 28 significant changes rank-ordered according to fold-change (Fig. 5b). Day 60 organoids showed an increase in volume (4×) and in ventricle size (2×) (Fig. 5c,d). An uneven expansion of cell population numbers (19X DN, 4X TBR1, 2X SOX2; Fig. 5d; Supplemental Fig. 5) produced a 56% reduction in SOX2^+^ frequency, doubling the TBR1:SOX2 ratio (Fig. 5e). A massive increase in DN cells was observed specifically in “TBR1-adjacent” regions and in the featureless “core” of the organoid. Cell type frequencies were generally consistent in replicate organoids, supporting the importance of whole-tissue analysis to reduce variability (Supplemental Fig. 5c).

Day 60 organoids contained a layer of DN cells separating SOX2 and TBR1 regions (Fig. 5a), which reduced the proximity of SOX2^+^ and TBR1^+^ cells to one another (43% decrease for SOX2^+^ and 34% decrease for TBR1^+^). These DN cells lead to a higher frequency of “TBR1^+^DN^high^” cytoarchitectures where abundant DN cells appeared above SOX2 cells and continued into the TBR1 region (Fig. 5f). These DN cells may represent the emergence of intermediate progenitors and non-TBR1 neurons consistent with cortical development. The overall production of new cells by SOX2 progenitors increased inter-ventricle and ventricle-to-surface distances in d60 organoids, which translated into 70% reduction in “Adjacent” and “Surface” cytoarchitectures. Development increased the frequency of “TBR1^+^DN^high^” and “TBR1^+^DN^low^” archetypal (TBR1^+^) cytoarchitectures. Nevertheless, these still only represent 50% of all radial patterns in the organoids. Older organoids also displayed an increase in “core DN” cells (14×) and a “DN only” cytoarchitecture (2×) oriented towards the organoid’s featureless core. Large DN regions and cell death are common in the core of large organoids given the limited transport of oxygen, nutrients and growth factors^37–39^.

We observed a relatively consistent distribution of cytoarchitecture frequency in our experimental replicates (Supplemental Fig. 5d). To confirm the consistency of cytoarchitectures, we computed SOX2 and TBR1 proximities of individual cells in the most abundant clusters: “Adjacent” in d35 and “TBR1^+^DN^high^” in d60 (Fig. 5g). “Adjacent” regions in younger organoids showed increased in TBR1 proximity ~ 75 µm from the ventricle then shifted back to SOX2 proximity when the virtual column captured a second population of SOX2 cells. In contrast, the more mature TBR1^+^DN^high^ regions showed a consistent transition from SOX2-proximity to TBR1-proximity ~ 150 µm from the ventricle.

### Comparative analysis of different cerebral organoid protocols

Cerebral organoid differentiation protocols vary in their cell diversities and inter-organoid heterogeneity. Recently, Velasco et al*.* and Yoon et al. have applied single-cell RNA sequencing to evaluate new dorsal forebrain organoid protocols showing improved consistency^12,22^. However, these studies have not quantified the variability of whole-tissue morphology within intact organoids. We compared d34 and d56 patterned dorsal forebrain organoids using the Velasco protocol, which uses WNT inhibition and TGF-β inhibition, avoids Matrigel droplet embedding and follows a different maturation schedule (Fig. 5h). Organoid development produced similar trends as our undirected protocol: increased cell numbers, larger tissues and larger ventricles (Fig. 5i–k). One important difference was the significant decrease in DN cells within these patterned organoids, which lead to a higher frequency of SOX2 cells and TBR1 cells in older organoids. Cytoarchitecture analysis produced a different but similar set of clusters in these patterned organoids (Supplemental Fig. 6). Nevertheless, development produced a similar shift towards TBR1^+^ cytoarchitectures with a decrease in “Adjacent” and “Surface” cytoarchitectures, consistent with increased differentiation of radial glia and the overall expansion of each ventricle/rosette region (Fig. 5l).

**Figure 6:**
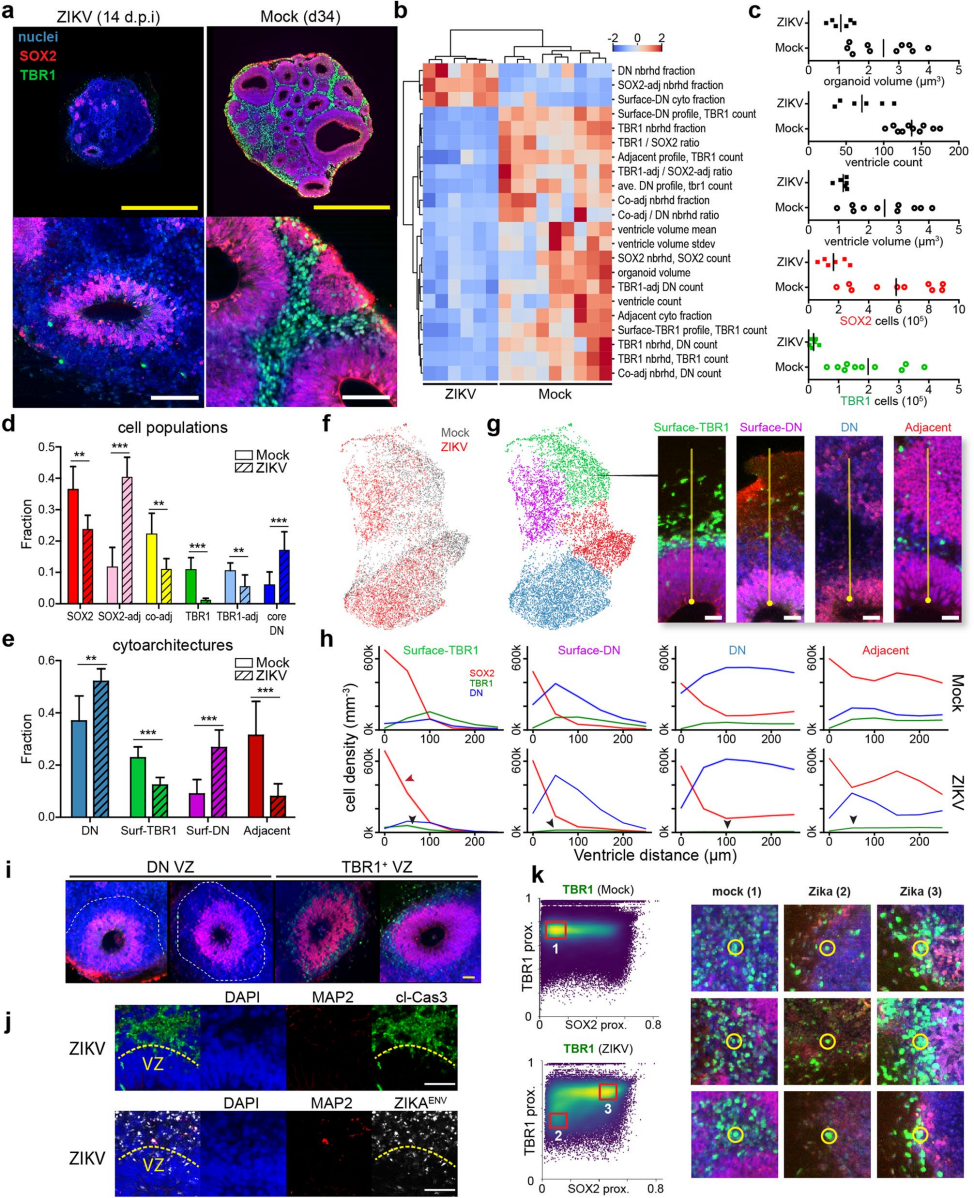
Hyper-dimensional analysis of multiscale pathology caused by Zika virus infection. (**a**) Representative image of age-matched day 34 mock and Zika virus-infected organoid (14 days post-infection) taken from volumetric datasets. Scale bar = 1 mm. (**b**) Heat map outlining major differences between nine ‘mock’ vs. six ‘Zika-infected’ organoids. (**c**) Dot plots showing organoid volume, ventricle count, ventricle volume, and total counts of SOX2 and TBR1 cells. (**d**) Comparison of cell subpopulation frequencies (***p < 0.001, **p < 0.01). (**e**) Comparison of cytoarchitecture frequencies (***p < 0.001, **p < 0.01). (**f**) UMAP embedding of the cytoarchitectures detected in mock (grey) and Zika-infected organoids (red) showing a general shift in cytoarchitecture. (**g**) UMAP embedding of cytoarchitecture clusters with representative images. Scale bar = 50 µm (**h**) Average profile plot of mock (top row) and Zika infected (bottom row) cytoarchitecture clusters showing the radial distribution of SOX2 (red), double negatives (blue) and TBR1 (green) cells. Arrow heads indicate the thinning of the SOX2 VZ (red) and the loss of TBR1 cells (black) in response to Zika infection. (**i**) Images of ventricles from Zika-infected organoids showing either a thick layer of DN cells or thin layer of TBR1 cells surrounding the SOX2 cells of the VZ. Scale bar = 50 µm. (**j**) Antibody staining of Zika infected organoids 14 dpi showing that the majority of cells outside the VZ are apoptotic (cleaved-Caspase-3 in green) and infected with Zika (Zika Envelope protein in white). (**k**) Left: Proximity scores of TBR1 cells from all mock- (top) and Zika-infected (bottom) organoids. Right: representative images corresponding to segmented cells in the regions selected the dot plots. Yellow circle shows the segmented cell. Scale bar = 50 µm.

As a proof-of-concept to evaluate SCOUT’s ability to discern multiscale differences between different protocols, we compared our day 60 organoids (protocol #1) with day 56 “Velasco protocol” (protocol #2) patterned organoids, (Fig. 5m-p, Supplemental Fig. 7, Video S3). SCOUT analysis detected 29 major differences (Supplemental Fig. 7b) including noticeably smaller organoids (− 60%) and ventricles (− 84%) in protocol #2. Patterned protocol #2 organoids also show a two-fold increase in SOX2 frequency and a three-fold increase in TBR1 frequency (Fig. 5m). SOX2 to TBR1 cell proximity increased in these organoids due to the low number of DN cells between the VZ and neurons. DN cells were less frequent overall, which produced a 70% reduction in DN cells specifically in the featureless core and in TBR1-adjacent regions.

As expected, cytoarchitectures were different in patterned protocol #2 organoids due to abundant TBR1 cells leading to appearance of a unique “TBR1-rich” cluster possessing increased TBR1 (+ 35%) and reduced DN counts (-70%) relative to the “TBR1 + DN^high^” cytoarchitecture in protocol #1 organoids (Fig. 5n–p). Higher occurrences of “Adjacent” and reduced “DN only” cytoarchitectures in patterned organoids (protocol #2) was consistent with their smaller size, which also lead to a 66% reduction in core DN cells. Patterned (protocol #2) organoids possessed better consistency across all scales, reducing variance in 72% of all SCOUT-computed features and improved the consistency of radial organization (Supplemental Fig. 7d–f).

Differences between these organoids can be due to numerous factors including the use of different cell lines, developmental trajectories, inhibitors, morphogens, culture timing and Matrigel administration. Nevertheless, this proof-of-concept analysis demonstrates the establishment of a comprehensive quantitative framework where different organoid models and culture protocols can be evaluated without bias. Image-based holistic analysis in combination with single-cell RNA sequencing and other techniques will facilitate the development of improved organoid models with reproducible single-cell and whole-tissue properties.

### Comparative analysis of Zika infection

Brain organoid characterization played an important role during the 2015–2016 Zika virus epidemic to model pathology in the developing brain. In utero exposure to Zika virus caused microcephaly, ventriculomegaly and cortical migrational abnormalities^40^ in newborns. Initial studies evaluated the virus susceptibility of various cell populations and the underpinnings of microcephaly^9,18,19,41,42^. These studies discovered altered VZ morphology and decreased production of mature neurons^9,18,41,43^. Building on this foundation, we applied SCOUT to quantify the multiscale impact of Zika virus infection on brain development in 3D datasets. We hypothesized that SCOUT pipeline would ensure detection of rare but important changes in tissue features and enable the quantification of previously unexplored features such as ventricle morphology, and comprehensive cytoarchitecture analysis. In particular, SCOUT can explore the multitude of tissue phenotypes in different subregions that arise from non-uniform virus infection and propagation.

Using a clinical isolate of Zika virus from Puerto Rico, we infected organoids at day 21 with a multiplicity of infection (MOI) of 0.1 and waited 14 days post-infection (dpi) to assess changes in tissue properties (Fig. 6, Supplemental Fig. 8 and Video S4). We detected 22 major differences with infection (Fig. 6b) including a ~ 50% reduction in organoid size, ventricle size and ventricle frequency (Fig. 6c, Supplemental Fig. 9). The loss of ventricles correlated with a 72% decrease in SOX2^+^ cells and a 75% decrease in “Adjacent” cytoarchitecture. Zika infection also changed the morphology of VZ with irregular and sparser arrangement of SOX2^+^ cells (Fig. 6a, Supplemental Fig. 9).

Since viral infection produced a ~ 90% decrease in TBR1^+^ cells (Fig. 6d), only rare VZs (1–2 per organoid) displayed a thin layer of TBR1 cells in Zika-infected organoids (Supplemental Fig. 10). Emergence of TBR1 cells in these specific subregions may have been due to lower virus exposure during infection or to faster recovery after the antiviral response. In contrast, the majority of ventricles contained a thin SOX2 layer lacking TBR1 cells almost completely, which is reflected in the predominance of “Surface-DN” (27%) and “DN only” (52%) cytoarchitectures and a four-fold increase in SOX2-adjacent DN cells (Fig. 6d,e,h). Interestingly, TBR1-negative ventricles contained a layer of DN cells thicker than the rare TBR1 layers in size-matched ventricles (Fig. 6i). Additional experiments revealed these DN cells to be virus-infected and apoptotic (Fig. 6j, Supplemental Fig. 10b).

The decreased TBR1^+^ neurons and increased DN cells shifted cytoarchitectures from predominantly “Adjacent” and “Surface-TBR1” in mock samples to “DN” and “Surface-DN” (Fig. 6f–h). Since these four cytoarchitectures occurred in both mock and infected organoids, we analyzed group-specific radial cell distribution profiles. TBR1 counts were reduced by 70% in Zika-specific instances of “DN”, “Surface DN”, “Surface TBR1” and “Adjacent” cytoarchitectures (Fig. 6h, arrows). “Surface-TBR1” profile analysis showed decreased width of SOX2^+^ cell distribution after infection, consistent with VZ thinning (Fig. 6h, red arrow). We confirmed a ~ 30% decrease in VZ thickness by computing the mean radial distance of SOX2 cells (Supplemental Fig. 9g).

Using SCOUT, we interrogated the spatial context of the rare TBR1^+^ cells (~ 1% of total cells) in Zika-infected organoids at single cell-resolution. In mock organoids, TBR1 cells cluster into densely populated layers above the VZ (population 1 in Fig. 6k). Zika infection caused TBR1 cells to either form thin layers adjacent to the VZ (high SOX2 proximity, population 3 in Fig. 6k) or appear sparsely in DN-rich regions at the surface (population 2 in Fig. 6k).

Overall, this analysis produced a first-of-its-kind comprehensive quantification of Zika-mediated pathology including: loss of cells, reduction of ventricles and overall tissue reorganization. We were able to characterize the spatial context of rare cells and to distinguish group-specific differences in cytoarchitecture. Infection phenotype reduced organoid size, ventricle growth, and the expansion of SOX2 and TBR1 cells (Supplemental Fig. 9). Given our observation that SOX2 cell counts correlate with multiscale tissue features (Fig. 4f), it is expected that Zika-related loss of neural progenitors produced a decrease in the complexity of tissue topography and cell patterning. Our characterization of the Zika virus phenotype is consistent with previous reports and may mirror the mechanisms of viral microcephaly in newborns. Unfortunately, it remains unclear whether the loss of TBR1^+^ neurons is due to disrupted progenitor differentiation or neuron-specific cell death. However, SCOUT can help discern between these two possibilities in future studies.

## Discussion

Here, we introduce SCOUT, a versatile platform that enables the automated analysis of single-cell, spatial, cytoarchitecture, and system-wide features. The combination of SHIELD tissue clearing, eFLASH antibody labeling and LSFM allows rapid acquisition of high-resolution three-dimensional datasets. Each organoid takes approximately 15 min to image with sufficient resolution to ensure accurate single-cell analysis. The automated extraction of ~ 300 multiscale features takes approximately 6 h per organoid. We demonstrated the power of SCOUT by quantifying multiscale feature correlation, maturation-related changes, protocol comparisons, and Zika virus pathology. SCOUT enabled the quantification of spatial features including cellular context, ventricle morphology, cytoarchitecture distribution and detection of rare events.

SCOUT analysis of Zika pathology highlights the importance of holistic multiscale analysis. Our findings are in line with previous reports: Cugola et al*.* showed a ~ 50% reduction in PAX6^+^ progenitors and TBR1^+^ cells at 4 dpi^41^; Qian et al. showed a ~ 33% reduction in ventricular zone thickness and a ~ 50% reduction in neuronal layer thickness at 14 dpi^9^. Previous studies also report a decrease in organoid size ranging from 20% at 14 dpi^18^ to 50% at 11 dpi^42^ and as high as 67% at 18 dpi^9^. These trends are generally consistent with some variation caused by distinct differentiation protocols, viral infection timelines, and histological subsampling.

Our pipeline provided unbiased quantification of cell population decreases upon Zika virus infection and discovered previously unreported reductions in ventricle volume, ventricle frequency, and a significant change in the spatial context of rare TBR1^+^ cells. Holistic analysis of multiple replicates enabled proximity analysis of rare TBR1^+^ neurons, which segregated into two populations. The thin layers of progenitor-adjacent TBR1^+^ neurons (P_i_^TBR1_high^ P_i_^SOX2_high^) are consistent with previous reports^9^. Howerver, the P_i_^TBR1_low^ P_i_^SOX2_low^ TBR1^+^ cells sparsely present in DN-rich regions near the organoid surface represent a new feature of Zika infection.

This initial study used two strategic antibodies in a single round of antibody staining to validate our analytical pipeline with ~ 50 organoids. Moving forward, it will be necessary to incorporate multiple rounds of organoid staining to increase the number of antibodies imaged and cell types analyzed. Increasing the number of markers imaged per round is challenging due to spectral overlap. The implementation of other strategies will be necessary to increase multiplexing such as barcoding of primary antibodies or multi-round tissue staining, both of which require precise co-registration of multiple 3D datasets with cellular precision to ensure accurate evaluation of marker expression at the single-cell level^29,44,45^. New markers should include cytoplasmic proteins, which can stain neuronal or glial projections. These cytoplasmic markers will require imaging at higher magnification and new computational approaches to achieve accurate segmentation of whole cells (not just their nuclei) in high-density regions. Both glial and neuronal cell projections often run in parallel over long distances and it remains a significant technical challenge to accurately ascribe these projections to their appropriate cell body or soma. To overcome this hurdle, it may be necessary to physically expand organoid tissues^44^ to increase image resolution sufficiently to disentangle adjacent projections or apply sparse labelling strategies^46^. In light of these challenges, our initial analysis centered on SOX2 and TBR1 nuclei to quantify 3D tissue architecture of brain organoids in the absence of a reference atlas.

At present, our pipeline’s single-cell and spatial context analysis can theoretically be used to analyze any tissue or cell population using the global expression/co-expression of two nuclear markers. In contrast, cytoarchitecture analysis relies on the successful segmentation of ventricle lumens and is likely limited to cerebral organoids. Nevertheless, in its current implementation, cytoarchitecture analysis can be used to quantify the arrangement of other radially-organized nuclear epitopes (e.g. TBR2^+^ intermediate progenitors or CTIP2^+^ neurons) in cerebral organoids using the automated generation of virtual cortical columns.

We employed widely available resources such as Docker and GitHub to make the SCOUT pipeline accessible for both immediate use and customization. All code and documentation for the SCOUT pipeline has been made open-source and available on GitHub for researchers to contribute, improve and expand. Changes can branch off the main SCOUT repository or can be amended to SCOUT via a pull request. This flexibility and support ensures SCOUT can be adapted to address new research questions. For researchers interested in using SCOUT without editing the source code, we have created a Docker image for executing the SCOUT pipeline on all major operating systems. Docker-based analysis still offers the ability to easily customize parameters for marker-based cell identification, spatial context analysis, and cytoarchitecture clustering. Docker installation also includes a tutorial for getting started with SCOUT using the pre-built Docker image (https://hub.docker.com/r/chunglabmit/scout) and a sample dataset for benchmarking.

The SCOUT pipeline provides an initial attempt at holistic cerebral organoid characterization. The lack of stereotypic development and a common coordinate system in organoids is problematic when using two-dimensional tissue sections for cellular and morphological analysis. SCOUT addresses this challenge by enabling a comprehensive 3D analysis of whole organoids. Using SCOUT, we quantified significant differences among the experimental groups with consistent trends among replicates. Unbiased high-throughput analysis of antibody-labeled organoids presents an important step in biological research since data analysis remains an important bottleneck in achieving organoid-based screening studies. Single-cell RNA sequencing is a powerful technique but cannot analyze more than a few thousand cells per organoid^5,12,47^. Other groups have made progress in 3D organoid imaging^20,21,48^ but lack comprehensive multiscale analysis in a computationally efficient pipeline.

SCOUT will enable future studies to analyze sufficient replicates to establish comprehensive quantitative phenotypes for different culture protocols and experimental perturbations. Improving the sensitivity of phenotype characterization facilitates the detection and quantification of subtle changes in organoid-based disease models. Our pipeline can also provide the necessary feedback to drive the development of new culture protocols achieving consistent three-dimensional landscapes with specific cell populations. We have already demonstrated how protocols can improve organoid consistency (Fig. 5). The combination of consistent tissues and comprehensive analytical pipelines will enable ambitious large-scale organoid-based screens to improve our understanding of developmental biology, its disorders, and potential treatment strategies.

## Material and methods

### Cerebral organoid culture

Organoids were cultured according to the protocol by Lancaster et al*.*^24^ using the SC101A iPS cell line (Systems Biosciences). The iPSC cells were cultured on Matrigel-coated (Corning) plates using mTeSR medium (Stemcell Technologies), passaged using ReLeSR at 80% confluency, and organoids were made before the 15th passage of initial cells. For organoids, single cells were detached with Accutase (Stemcell Technologies) when iPS cells were at 60–80% confluency. We seeded 9,000 cells in ultra-low attachment round bottom 96-well plates in hESC medium^24^ with 4 ng/mL bFGF (Peprotech) 50 µM Y-27632 Rock-inhibitor (Tocris) for the first 4 days then without for an additional two days. At day 6, organoids were transferred to ultra-low attachment 24-well plates in neural induction medium^24^ with addition of SMAD inhibitors^16,49^ 10 µM SB-431542 and 1 µM dorsomorphin (Tocris). At day 9, neural induction medium was replaced with fresh medium without the SMAD inhibitors. At day 12, organoids were embedded in 15µL growth-factor reduced Matrigel droplets (Corning). We cultured 12 organoids per 60 mm Petri dish for suspension culture in 5 mL cerebral organoid differentiation medium^24^ without vitamin A for 4 days. At day 16, medium was replaced with cerebral organoid differentiation medium with vitamin A^24^ and placed on shaker at 85 rpm. Medium was replaced twice per week and at day 40, we added 14 ng/mL BDNF to organoid medium^5^. The dorsal forebrain “Velasco et. al" organoids used in the comparative analysis of protocols were derived from the Mito 210 iPSC line (from the laboratory of Bruce Cohen, McLean Hospital), as previously described^12^.

### SHIELD sample preparation

Organoids were rinsed once with PBS, then fixed with freshly prepared 4% PFA in PBS (EM grade, Electron Microscopy Sciences) at room temperature for 30 min on a shaker. Organoids were rinsed three times in PBS, transferred into ice cold supernatant of 2% polyglycerol 3-polyglycidylether (wt./v) in 0.1 M phosphate buffer pH 7.2 and incubated for two days at 4 °C. Organoids were subsequently transferred into pre-warmed 0.1 M sodium carbonate buffer (pH 10) and incubated at 37 °C for 24 h. Organoids were washed extensively with PBS for 8 h, cleared in 0.2 M SDS buffer for 48 h at 55 °C while shaking in EasyClear system (LifeCanvas Technologies), and washed extensively in PBST (PBS, 0.1% Triton X-100, 0.02% sodium azide) for 24 h.

### mRNA fluorescent in-situ hybridization (FISH)

GFP-labeled organoid was prepared by labeling day 35 organoids with a 1/500 dilution of human adenovirus type 5 expressing eGFP under control of a CMV promoter (Vector Biolabs; Ad-GFP). Three days after viral labeling, organoids were fixed and SHIELD-processed in supernatant of 2% P3PE solution, (as described above) but without clearing. Organoids were then embedded in low melt agarose and sliced at 200 µm thickness by vibratome (VT1000S, Leica Biosystems, Germany). Organoid slices were subject to passive clearing using the 0.2 M SDS, 50 mM phosphate (pH7.3) clearing buffer at 37C for 24 h and extensively washed by PBST. Fluorescence in situ hybridization-hybridization chain reaction (FISH-HCR) of GFP was performed as described before^26^ using 50nt GFP probes and B1 Alexa 647 hairpin. FISH-stained sample was imaged by an Olympus confocal microscope (FV1000MPE) with 20X 1.0 NA water objective (XLUMPlanFL N).

### ZIKV production and infection

ZIKV strain PRVABC59 (Puerto Rico, ZIKV^PR^) was obtained from ATCC and expanded in C6/36 mosquito cells. To establish tittered viral stocks, virus-containing supernatant was harvested and viral titer was determined by the focus forming unity assay using BHK cells^50^. To infect cerebral organoids, 1e5 FFU/organoid was added to culture media on a shaker. Viral inoculum was washed off and replaced after 24 h with fresh medium. Organoids were infected at day 21, cultured at 85 rpm. as usual and analyzed 14 days later.

### eFLASH sample preparation

SHIELD-processed and cleared organoids were stained using an adapted version of the eFLASH protocol^27,44^. We incubated organoids in eFLASH sample overnight at room temperature. Organoids were then placed in the SmartLabel system (LifeCanvas) with 1.4 mL sample buffer in the sample cup. For SCOUT pipeline, we added 6µL Syto16 (1 mM solution, ThermoFischer #S7578), 15 µg goat anti-SOX2 antibody (R&D Systems #AF2018), 10 µg Fab fragment anti-goat IgG Alexa Fluor 594 (Jackson ImmunoResearch #805-587-008), 30 µg rabbit anti-TBR1 Alexa Fluor 647 (Cell Signaling Technology #45664S). Additional antibodies used for whole organoid staining are anti-β3-tubulin (mouse monoclonal, Biolegend #657408), anti-MAP2 (mouse monoclonal, Biolegend #801803) and vimentin (rabbit monoclonal, Cell Signaling Technologies #45664). All antibodies used in this study are listed in Supplementary Table 1.

### Whole-organoid imaging

Prior to imaging, organoids were equilibrated in PROTOS-based immersion medium^27,29^ in two steps. First, we incubated organoids in a 1:1 mix of PROTOS and PBS for 4 h. Then, we replaced the solution with PROTOS immersion medium for at least 6 h. Images in Fig. 1 were acquired using the Leica TCS SP8 laser-scanning confocal microscope with a white light laser source for excitations at 488, 594 and 647 nm using a 20X 0.5-NA water-immersion objective (Leica #15506147, HCX APO L 20x/0.50 W U-V-I). For rapid volumetric imaging in subsequent figures, PROTOS-immersed samples were mounted in a 1.5% agarose prepared by warming agarose in PROTOS with a microwave. We were able to mount 6–8 organoids per block at a time. After agarose polymerization, the block was equilibrated in 25 mL PROTOS overnight. Samples were mounted and imaged with a SmartSPIM axially swept light-sheet microscope (LifeCanvas Technologies) equipped with three lasers (488 nm, 561 nm, 642 nm) and a 10× objective (Olympus XLPLN10XSVMP, 0.6NA, 8 mm WD, lateral resolution 0.65um in XY). We imaged samples at a 0.65 × 0.65 × 2 µm voxel size. In all of our experiments presented, we used replicate organoids (n) from the same batch.

### Preprocessing LSFM images of cerebral organoids

Cerebral organoid images from the LSFM system (LifeCanvas Technologies, SmartSPIM) originally stored in an uncompressed binary format were first destriped and stitched according to a previously reported image processing pipeline^51^. This pipeline generates z-slice images with lossless compression that are used to compute image histograms for each channel. After normalizing each channel to the 99th percentile of the histogram, each channel is then partitioned into (64, 64, 64) voxel chunks using the Zarr Python package. This chunk-compressed representation allows for parallel processing of each image data chunk.

### 3D nuclei detection using curvature-based seeded watershed

To detect all nuclei in volumetric nuclear stain images, a curvature-based nuclei filtering strategy was developed. An image filter based on the eigenvalues of the Shape Operator (also known as the Weingarten matrix) was created by defining a probability distribution over the image curvature and intensity as evidence for a nucleus centroid (Supplementary Fig. 2). This nucleus probability map highlights nuclei even in densely-packed regions such as the ventricular zone in cerebral organoids. The probability map was computed for each image chunk after Gaussian smoothing to remove noise. Nuclei centroids were extracted from these probability maps through local maxima detection. The optimal parameters for Gaussian smoothing that maximize the detection F_1_-score were determined by validation with a set of 150 labeled nuclei centroids. To segment each nucleus, the detected nuclei centroids were used as seed points for performing a watershed segmentation of a nucleus foreground mask. This nucleus foreground mask was obtained through binarization of the nucleus probability map. Binarization of the nuclei probability map was accomplished by applying a threshold.

### Nuclei detection accuracy measurement

To assess the accuracy of our nuclei detection strategy, nuclei centroids were hand-annotated using a previously reported image alignment tool called Nuggt. 1,237 nuclei centroids were hand-annotated in organoid subregions including the ventricular zone and subventricular zone. Detected nuclei were considered true positives (TP) if they fell within 5 voxels of a ground-truth centroid and false positives (FP) if not. Since multiple detections may lie within 5 voxels of a given ground-truth centroid, double-counting TPs was avoided by matching ground truth and detected centroids using the Hungarian method for solving the linear sum assignment problem. The result of this matching procedure are lists of all TPs, FP, false negatives (FN), and true negatives (TN). These accuracy statistics were used to compute the overall accuracy and F1-score of the nuclei detection strategy. Our curvature-based seeded watershed algorithm was benchmarked against the difference of Gaussian (DoG) and Laplacian of Gaussian (LoG) blob detection algorithms implemented in the scikit-image Python package (Supplementary Fig. 2).

### Cellular subcategorization using in situ cytometry and spatial proximity analysis

Protein expression of transcription factors was measured by sampling the SOX2 and TBR1 antibody staining in a 3-voxel diameter spherical ball centered around the nuclei centroids. The mean fluorescence intensity within each sphere was computed for each channel and gated to define SOX2-/+ and TBR1-/+ cell populations. The result of this gating strategy is a labeled point cloud of nuclei centroids.

Spatial proximity analysis of the labeled nuclei point cloud was accomplished by first constructing a KD-tree representation of the point cloud for efficient querying of nearest nuclei. The spatial proximity to the nearest $n$ cells of type $t$ was calculated using the following formula for each detected nucleus $i$:

$$P_{i}^{\left( t \right)} = \mathop \prod \limits_{j = 1}^{n} \frac{1}{{1 + d_{i,j} /\sigma^{\left( t \right)} }}$$

where ${d}_{i,j}$ is the distance between the $i$-th nucleus and the $j$-th nearest nucleus and ${\sigma }^{(t)}$ is a reference distance that controls the proximity bandwidth (how close a neighboring nucleus must be to be considered in close proximity).

### Automatic 3D ventricle segmentation using U-Net

We adapted U-Net^35^, a convolutional neural network, to detect SOX2-lined ventricle lumens based on nine manually segmented whole-organoid datasets containing 7596 nuclear dye images (Supplementary Fig. 3). We produced training datasets of segmented SOX2-lined ventricles using ITK-SNAP^52^ to volume-fill ventricle lumens apparent in the SOX2 antibody channel. Segmented ventricle images were converted to binary and down-sampled. These images were combined with their corresponding Syto16 images at the same resolution and split with a 20% hold-out test set before moving on to model training and validation. The remaining training set was used with ten-fold cross-validation to tune model hyperparameters and the overall model architecture. We trained U-Net to detect ventricles using the nuclear dye images to potentially eliminate the necessity of SOX2-antibody in future studies. Automated ventricle segmentation by U-Net achieved a Dice coefficient of 97.2% on the holdout test set.

The U-Net model was implemented in Keras and slightly modified from the original architecture. Since our images were higher resolution than what was used in the original U-Net paper, we added two layers before and after the U-Net bottleneck to increase the receptive field of the model. This modified U-Net model was trained using a hybrid loss containing a weighted binary cross entropy (WBCE) term and a Dice coefficient loss term. The WBCE term was weighted at 90% to the Dice coefficient loss term's 10%. In our experience, the WBCE term helps the model converge to sensible ventricle segmentations due to a simpler gradient signal during training. However, the Dice coefficient loss is required to compensate for the high degree of class imbalance in any given training image.

The test accuracy was assessed after all training and validation steps were completed by computing the Dice coefficient for all test images (Supplementary Fig. 3). The receiver operating characteristic curve was constructed using a random sample of 100 images from the test set to speed up computations. These test images were segmented and thresholded at linearly spaced probability values between 0 and 1. For each threshold, the TP, FP, and FN rates were computed from corresponding pixels in the predictions and ground truth images. These rates were used to compute the final precision and recall values as well as the maximum F1-score and area under curve (AUC).

### 3D cytoarchitectural analysis of cerebral organoids

Binary ventricle segmentations were converted into a 3D mesh using the ‘marching cubes algorithm’ from the scikit-image Python package. The resulting mesh contained vertices, faces, and normal vectors uniformly distributed over the ventricle surfaces. The normal vectors and vertices (virtual cortical columns) from the 3D mesh were then used to query which nuclei centroids were within a 50 µm diameter and 300 µm tall cylindrical volume around the surface normal. These cells were bin counted for each cell type over 50 µm intervals to construct the final radial cell profiles.

To determine cytoarchitectural types, 5000 radial cell profiles were sampled from each organoid in a given dataset (i.e. from both day 35 and day 60 organoids in the maturation analysis in Fig. 5). These radial cell profiles were flattened into vectors and concatenated into a matrix of cytoarchitecture observations and features. This matrix was provided to UMAP to visualize the distribution of cytoarchitectures in 2D and perform dimensionality reduction before clustering. After performing UMAP embedding, the UMAP model was saved for future analysis. The UMAP embedded cytoarchitectures were then grouped using hierarchical clustering with a Euclidean distance metric and average linkage method. The cluster labels were saved and used as training data in a nearest neighbor cytoarchitecture classifier. Using the saved UMAP model and the pre-trained nearest neighbor cytoarchitecutre classifier, all profiles in each organoid were classified efficiently. The resulting cytoarchitectural labels were used to compute average profiles for each cluster.

### 3D rendering of ventricles

The 3D rendering process involves taking intermediate results in the SCOUT pipeline, including the ventricle segmentation and detected cell coordinates, and exporting them in a format that can be imported into Blender, a free program for 3D rendering and animation. Blender can be obtained from https://www.blender.org/ and we used version 2.8 for all of this work.

After U-Net ventricle segmentation, a polygon mesh of the ventricles is computed using the marching cubes algorithm in scikit-image, which results in a set of ventricle vertices and surface normal vectors that are saved in .OBJ format. We have provided a utility function called cyto.write_obj() which will create these OBJ files. These OBJ files can be directly imported into Blender, resulting in a 3D model of the organoid ventricles. For ventricle surface normals, we create a particle system of small cylinders positioned at the same vertices in the OBJ mesh and only render approximately 10% of all normals in the mesh. Single-cell point clouds are exported into Blender in a similar way, but the OBJ files contain the (x, y, z) coordinates of each detected cell centroid. Once imported, the mesh will have a default gray color, and different materials can be added to provide different colors on the ventricle surface. After performing clustering of radial profiles, a CSV file is written that indicates the type of radial profile each ventricle normal corresponds to in the SCOUT analysis. The SCOUT documentation contains a Python script that can be executed within the Blender script editor to read these labels and apply unique materials for each cluster label. This allows the ventricle surface to be colored according to the cytoarchitecture observed at each point on the ventricle surface.

We have included all the code and a summary of the steps involved in creating the 3D renders presented in Fig. 3 in the SCOUT documentation and user guide, which is available at:https://chunglabmit.github.io/scout/cytoarchitecture.html#d-rendering-with-blender.

### Hyperdimensional statistical testing for comparative organoid studies

To perform statistical testing on all multiscale features, we used independent two-tailed t-tests to obtain significance values for each feature. To reduce the number of false positives due to multiple comparisons, we also thresholded the significant phenotypic changes at a two-fold change in mean. This thresholding restricted the detected hits to those with large changes in mean so that those significant differences that are due to small sample standard deviations are removed.

### Pairwise correlation analysis of multiscale organoid features

For 12 day 35 organoids, the Pearson correlation coefficient and associated P-values were computed for all pairs of organoid features. These correlation coefficients were stored in a matrix and bi-clustered using hierarchical clustering. To construct a network from these pairwise correlations, the absolute values of the correlation coefficients were thresholded at 0.75 to remove edges in the network with smaller weights. This thresholded matrix of pairwise correlation coefficients was used as an adjacency map in the NetworkX Python package. The resulting network was then visualized in an interactive plot using the pyvis Python package. All heatmaps were generated using Matplotlib 3.1.2 (https://matplotlib.org/) and Seaborn 0.9.0 (https://seaborn.pydata.org/).

### Estimation of intra-organoid section variability using pseudo-sections

To compare the intra-organoid variability of sections to the inter-organoid variability in 3D analysis, the nuclei centroids and cell-type labels from 10 day 35 organoids were used in both 2D and 3D analyses. Pseudo-sections were 100 µm virtual sections of the same underlying 3D dataset of nuclei centroids and cell-type labels that align with the XY plane and fall within the Z limits of the organoid. 10,000 pseudo-sections were sampled from each organoid and used to compute the overall (normalized) cell frequency for SOX2, TBR1, and DN cells. Distributions of these pseudo-section cell-type frequencies were compared to the distributions of cell-type frequencies obtained by comparing the 3D organoid datasets directly.

## Supplementary information

Supplementary Figures.

Supplementary Table 1.

Supplementary Table 2.

Supplementary Information.

Supplementary Video Legends.

Supplementary Video 1.

Supplementary Video 2.

Supplementary Video 3.

Supplementary Video 4.
