# Supplementary material for: Multiscale 3D phenotyping of human cerebral organoids
Source: Sci Rep. 2020 Dec 8;10:21487. doi: 10.1038/s41598-020-78130-7 (PMC7723053; doi:10.1038/s41598-020-78130-7)
Supplement: Supplementary file 1 — Supplementary Figures. [file 41598_2020_78130_MOESM1_ESM.pdf]

Multiscale 3D Phenotyping of Human Cerebral Organoids

Alexandre Albanese[1,2,11], Justin M. Swaney[3,11], Dae Hee Yun[1,2,4] Nicholas B. Evans[1,2], Jenna M. Antonucci[1], Silvia Velasco[5,6], Chang Ho Sohn[1,2], Paola Arlotta[5,6], Lee Gehrke[1,7,8], Kwanghun Chung[1-4,9,10,*]

[1] Institute for Medical Engineering and Science, MIT, Cambridge, MA, USA

[2] Picower Institute for Learning and Memory, MIT, Cambridge, MA, USA

[3] Department of Chemical Engineering, MIT, Cambridge, MA, USA

[4] Department of Brain and Cognitive Sciences, MIT, Cambridge, MA, USA

[5] Department of Stem Cell and Regenerative Biology, Harvard University, Cambridge, MA, USA.

[6] Stanley Center for Psychiatric Research, Broad Institute of MIT and Harvard, Cambridge, MA, USA

[7] Department of Microbiology and Immunobiology, Harvard Medical School, Boston, MA, 02115

[8] Harvard–MIT Program in Health Sciences and Technology, Cambridge, MA, 02139

[9] Center for Nanomedicine, Institute for Basic Science (IBS), Seoul, Republic of Korea.

[10] Yonsei-IBS Institute, Yonsei University, Seoul, Republic of Korea.

[11] authors contributed equally

[*] Correspondence should be addressed to K.C. (khchung@mit.edu)

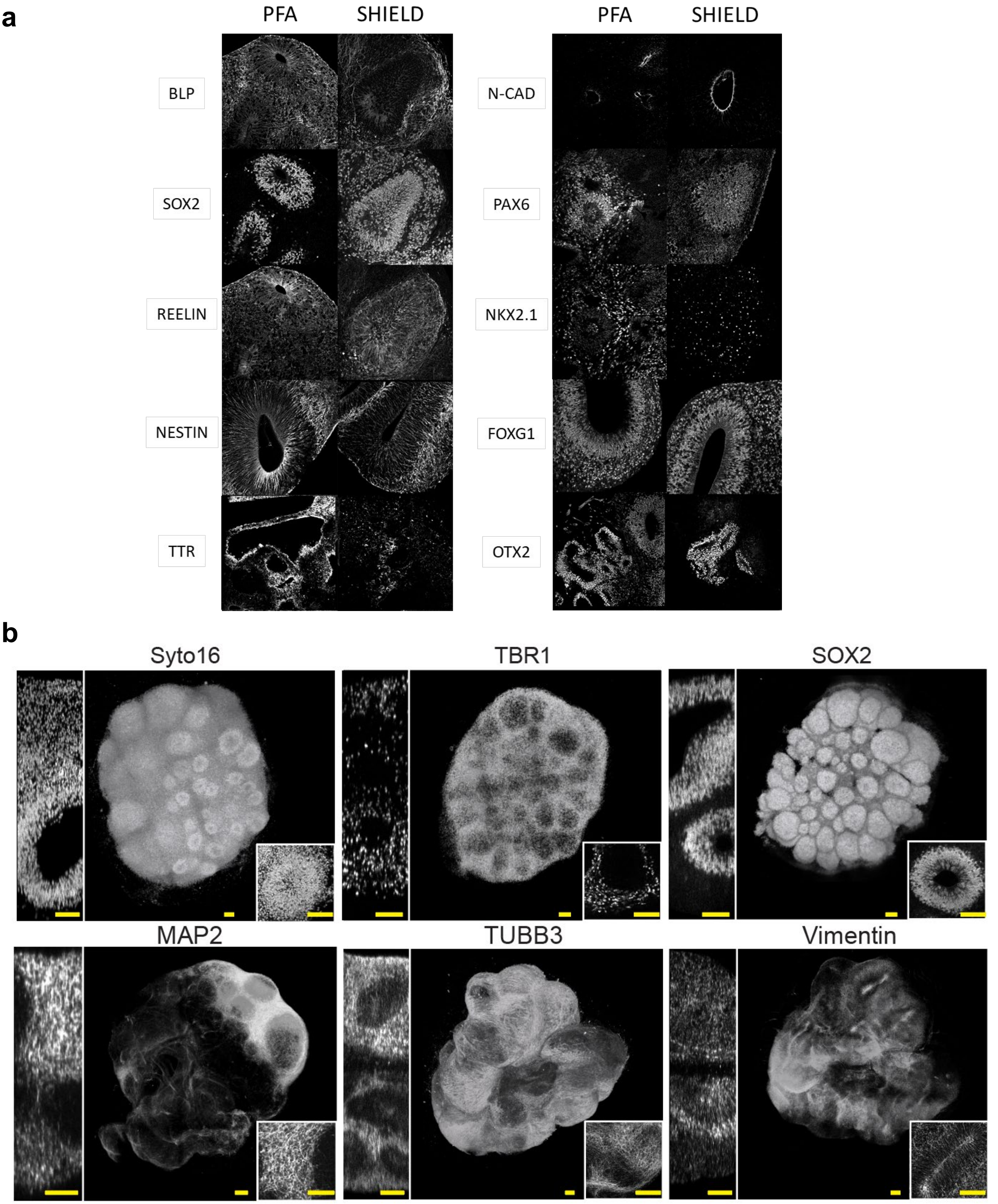

Supplementary Figure 1: Epitope Preservation of Major Markers: (**a**) Organoids were fixed with 4% paraformaldehyde (PFA) for 30 min at room temperature. A subset of organoids were then processed and cleared using the SHIELD protocol (see methods). Various antibodies to common cerebral organoid markers were tested (Supplementary Table 1). SHIELD-based tissue clearing did not affect the staining of antibodies tested (**b**) Day 35 whole-organoid staining of different antibodies using eFLASH. XZ plane (left of each panel) shows consistent labeling throughout the tissue and insets (bottom right of each panel) shows single-cell staining. Scale bars = 100 µm.

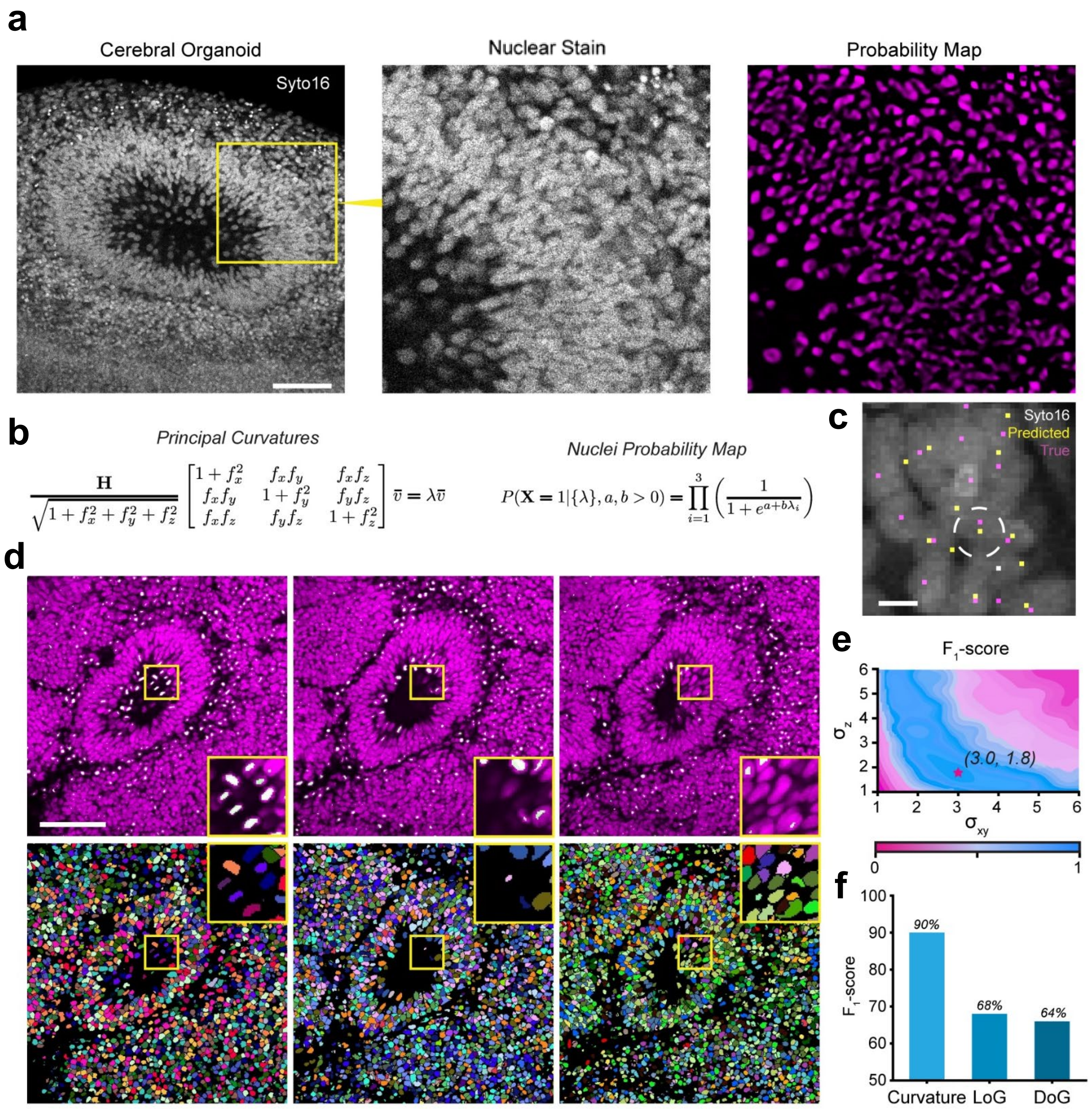

Supplementary Figure 2: Nuclei detection and segmentation accuracy: (**a**) Nuclei detection and semantic segmentation of a Syto16-stained cerebral organoid. The nuclei seed points are used in a 3D watershed segmentation to partition the foreground mask among all the nuclei detected as peaks in the probability map. Scale bar, 100 µm. (**b**) Analytical definitions of the shape operator and logistic regression function used to calculate nuclei principal curvatures and probability maps, respectively. (**c**) 2D slices and nuclei segmentations for a Syto16-stained cerebral organoid. Scale bar, 100 µm. (**d**) Nucleus detection accuracy assessed by matching with manually annotated nuclei centroids. Scale bar, 10 µm. (**e**) Tuning hyper-parameters for Gaussian filtering by maximizing F1 score. (**f**) Test set F1 scores from our technique compared to Laplacian (LoG) and Difference (DoG) of Gaussian blob detectors, which are widely used for segmentation.

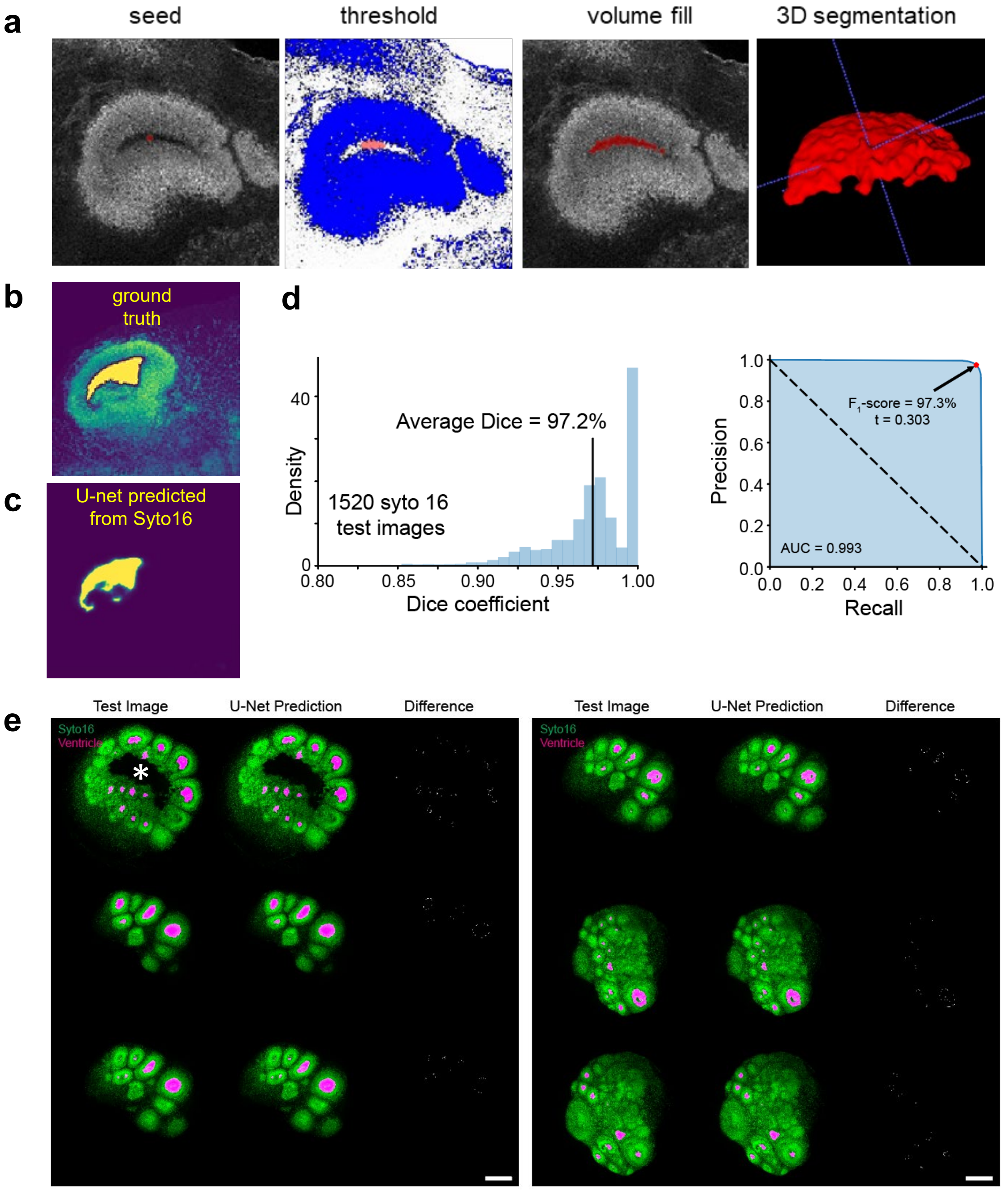

Supplementary Figure 3: Ventricle segmentation: (**a**) Example workflow for semi-automatic active-contour segmentation of ventricles using ITK-Snap. A spherical seed volume is iteratively dilated within the masked region obtained from thresholding the SOX2 image. After dilation, the ventricle lumen is "volume filled" up to thresholded SOX2 cells and the segmentation can be visualized in 3D using ITK-Snap (X, Y, Z axes in purple). (**b**) Overlay of a 2D Syto16 image with the associated ventricle segmentation from the ground-truth 3D segmentation. (**c**) Example ventricle probability map output from our U-Net model. These 2D probability maps contain floating point values between 0 and 1. (**d**) Test performance of ventricle segmentation using U-Net based on a 20% hold-out test set (1520 images). The Dice coefficient was computed for each test image resulting in an average of 97.2%. By sweeping the probability threshold used to convert the U-Net output probability maps into binary masks from 0 to 1, the model precision and recall were calculated to construct the ROC curve. (**e**) Representative images from two test sets showing the nuclear staining in green, the U-Net predicted ventricle in magenta and the difference between the prediction and manual annotation in white. Asterisk shows a hollow necrotic core, which is not segmented as a ventricle by U-net.

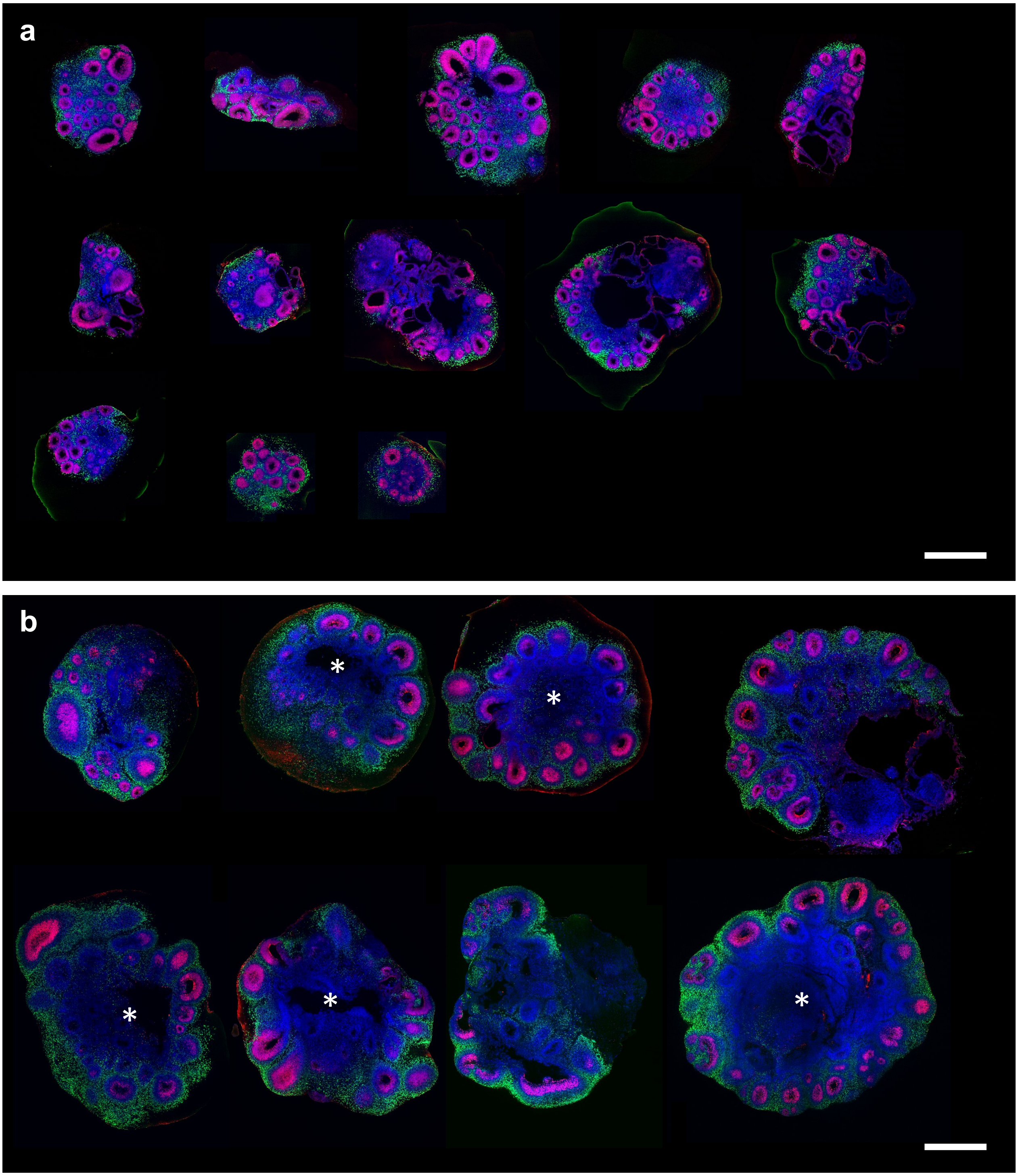

Supplementary Figure 4: Organoid maturation experiment: Middle pseudo-slice of day 35 organoids (**a**) and day 60 organoids (**b**) used in Figure 5 analysis. White scale bar = 500 µm. Pseudo-slice colors represent Syto16 (blue), SOX2 (red) and TBR1 (green). Asterisks indicate the necrotic core, which became empty during tissue processing. The necrotic core is surrounded by double negative cells.

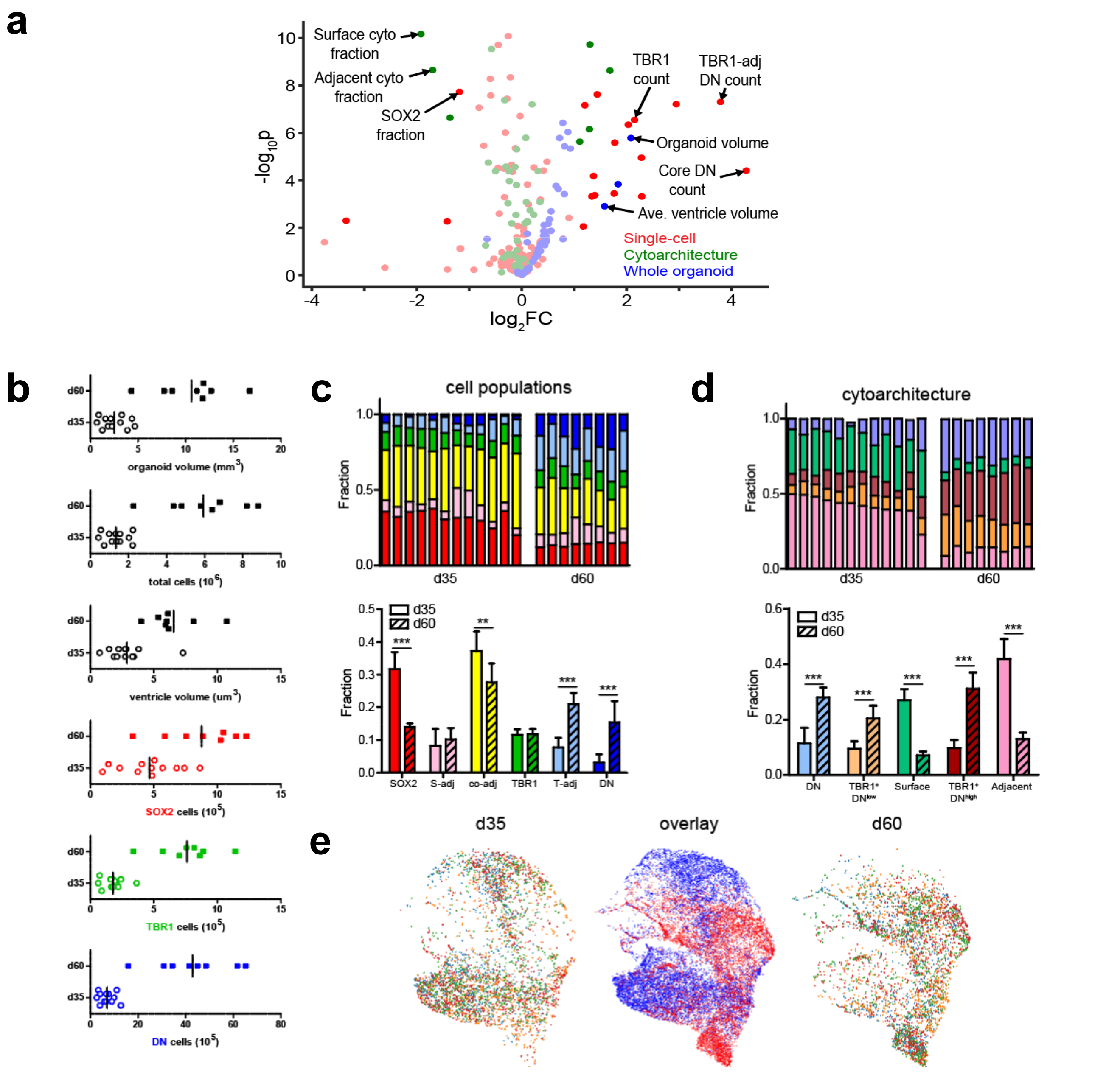

Supplementary Figure 5: Undirected organoids d35 vs. d60 comparison: (**a**) Volcano plot highlighting unbiased detection of multiscale differences with dots colored according to scale of analysis. (**b**) Dot plots showing organoid volume, total cells, average ventricle volume, and total counts of antibody-labeled subsets. (**c**) Cell subpopulation frequencies for independent replicates (top) and average values (bottom). (*** p<0.001, ** p<0.01) (**d**) Quantitative analysis of cytoarchitecture frequencies for independent replicates (top) and average values (bottom). (*** p<0.001). (**e**) UMAP embedding of the cytoarchitectures detected in day 35 and day 60 organoids. Left shows the distribution of cytoarchitectures in day 35 organoids where each color represents a different organoid. Middle UMAP shows cytoarchitectures according to the age of organoids (day 35 blue and day 60 red). Right UMAP shows the distribution of cytoarchitectures in the day 60 organoids where each color represents a different organoid.

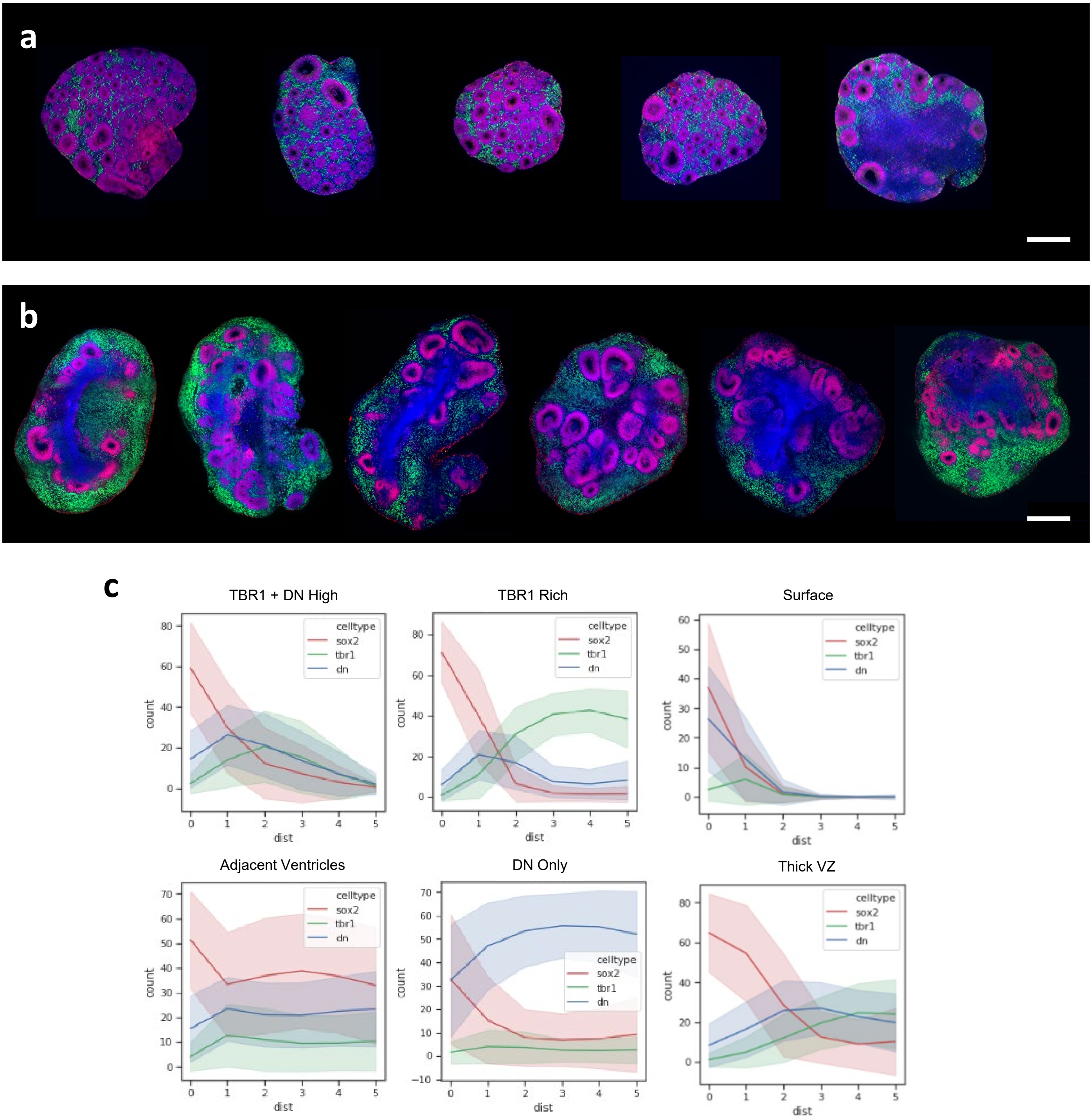

Supplementary Figure 6: Cytoarchitecture clusters for Velasco d34 vs. d56 comparison. Middle pseudo-slice of day 34 (**a**) and day 56 (**b**) organoids prepared according to Velasco *et al*. used in Figure 5 analysis. Scale bar = 500 µm. Pseudo-slice colors represent Syto16 (blue), SOX2 (red) and TBR1 (green). (**c**) Average radial distribution of different cell populations with standard error (transparent fill) for the different cytoarchitecture clusters used specifically in the analysis of patterned organoids.

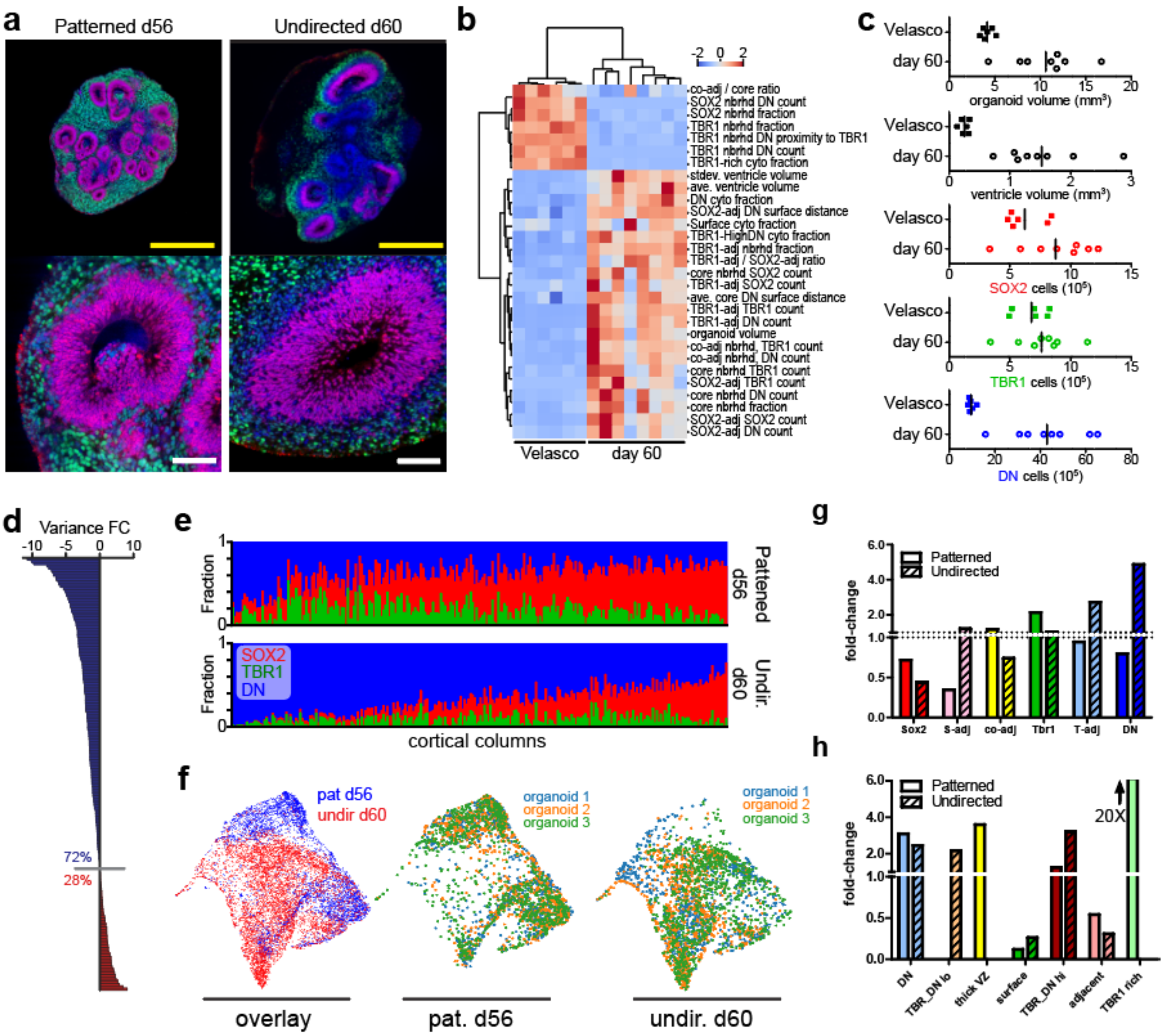

Supplementary Figure 7: Hyper-dimensional analysis of different culture protocols. (**a**) Representative image of day 56 Velasco and our day 60 organoids taken from volumetric datasets. (yellow bar, 1 mm; white bar, 100 μm) (**b**) Heat map outlining major differences between different organoid culture protocols. (**c**) Dot plots showing organoid volume, average ventricle volume, and total counts of cell types. (**d**) Comparative analysis of the fold-change in the relative standard error of features in Velasco organoids relative to undirected day 60 organoids. Dotted line shows zero-fold change. Blue bars reflect better homogeneity in patterned Velasco organoids (reduced error) in 72% of features. Red bars show higher standard error in 28% of features. (**e**) Frequency of SOX2 (red), TBR1 (green) and DN (blue) for 250 random virtual cortical columns pooled from all organoids. (**f**) Left shows UMAP embedding of the cytoarchitectures detected in Velasco (blue) and day 60 organoids (red). Middle UMAP shows cytoarchitectures collected from three Velasco patterned organoids. Right UMAP shows cytoarchitectures from three undirected day 60 organoids. (**g**) Comparison of fold-change in cell subpopulation frequency after maturation in the undirected (d 35 vs. d60) and patterned (d34 vs. d56) organoids. (**h**) Comparison of fold-change in cytoarchitecture frequency after maturation in the undirected (d 35 vs. d60) and patterned (d34 vs. d56) organoids.

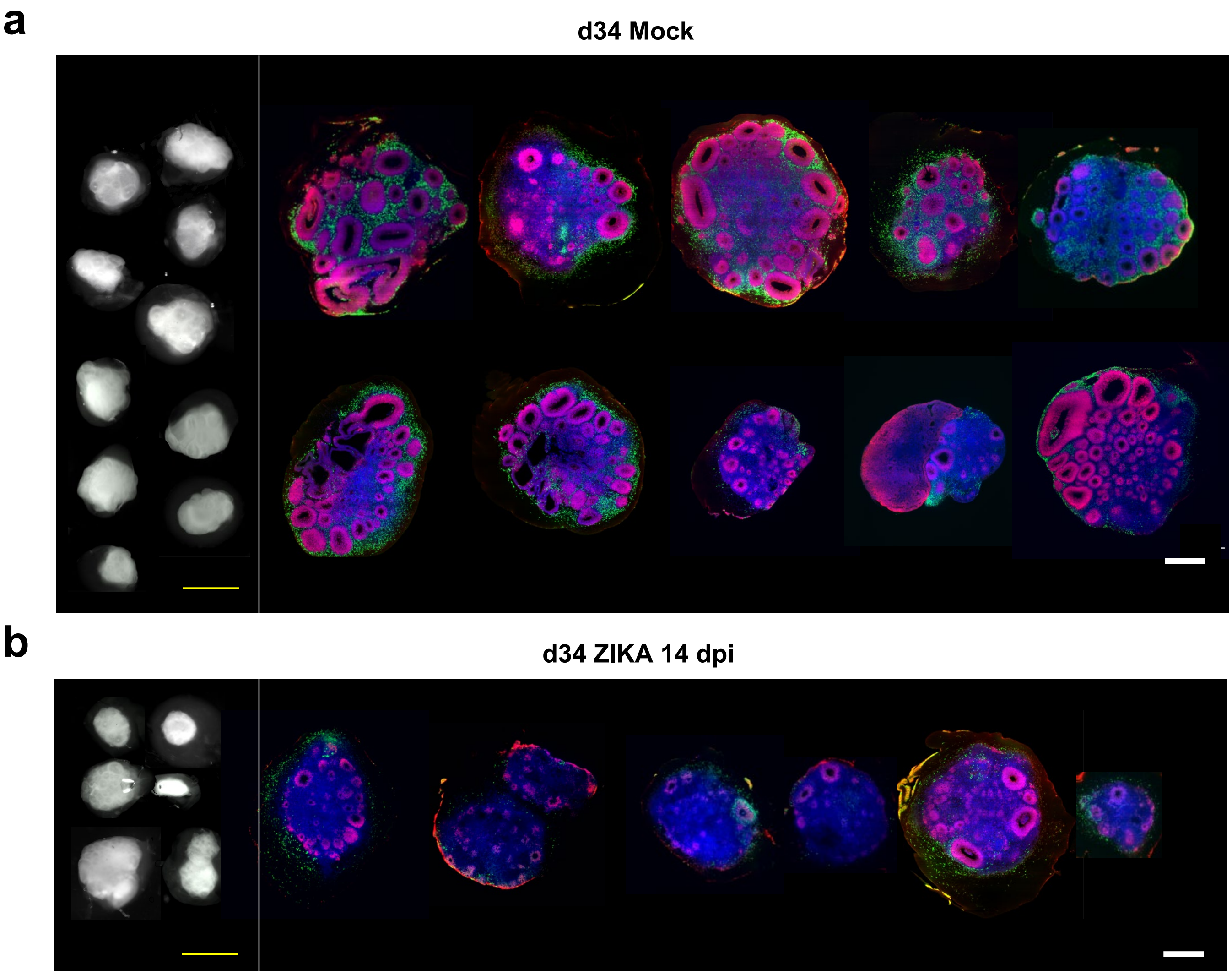

Supplementary Figure 8: Zika infection organoids : Dark-field images and middle pseudo-slice of “mock” organoids (**a**) and Zika-infected organoids (**b**) used in Figure 6 analysis. Yellow scale bar, 2 mm; white scale bar, 500 µm. Pseudo-slice colors represent Syto16 (blue), SOX2 (red) and TBR1 (green). (**c**) Examples of cell patterns around the ventricles of Zika virus-infected organoids.

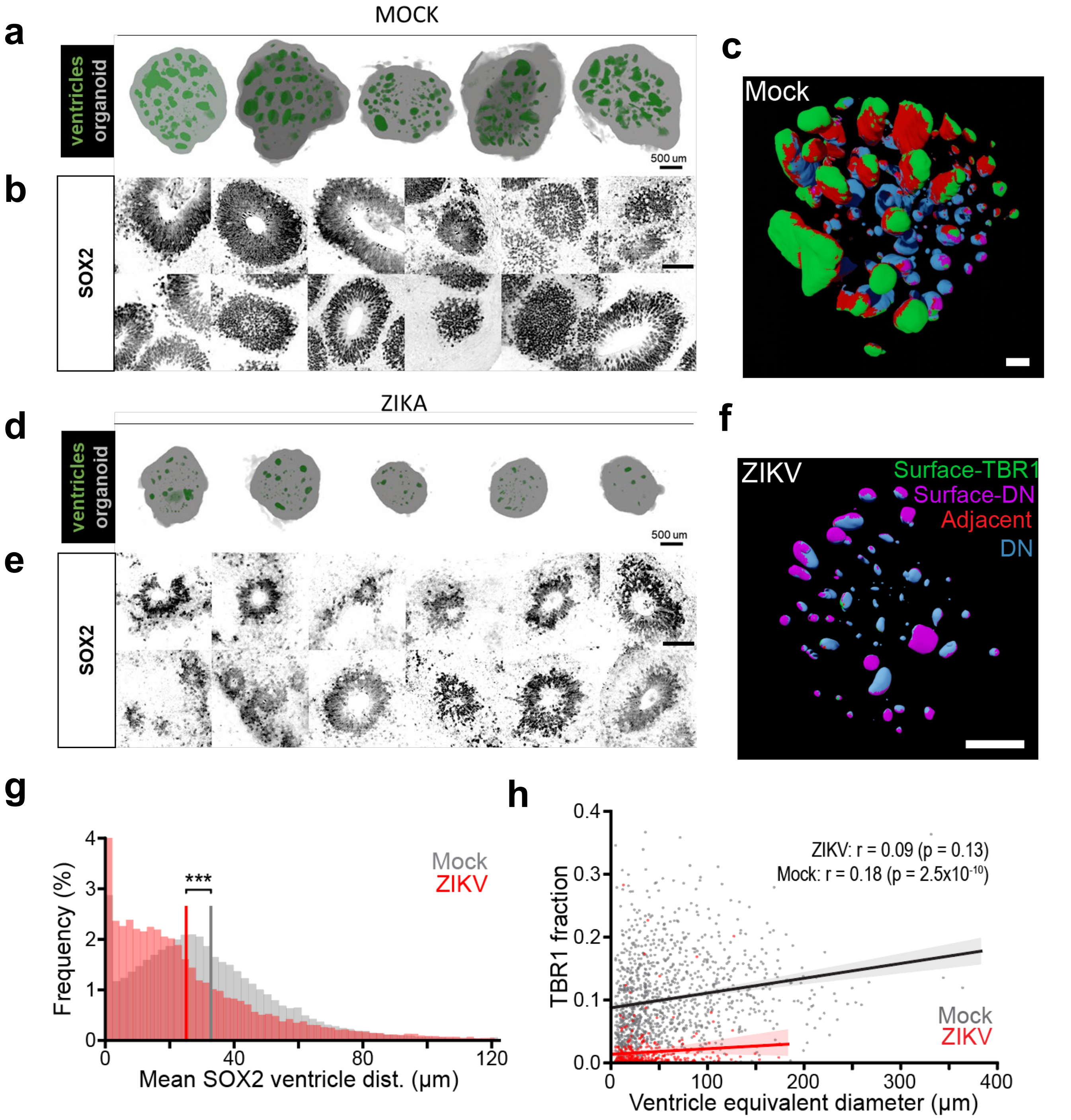

Supplementary Figure 9: Zika virus effect on ventricles and ventricular zones: (**a**) 3D renders showing ventricle segmentation (green) and organoid foreground segmentation (grey) of mock organoids. (**b**) Selected images from "mock" organoids showing the general morphology of ventricular zones. Images taken from anti-SOX2 staining and inverted. Scale bar = 100 µm. (**c**) Rendering of segmented ventricles in a Mock organoid with their surfaces colored according to detected cytoarchitecture. Clusters are represented using the same colors as Fig 6e and g. Scale bar = 200µm. (**d**) 3D renders showing ventricle segmentation (green) and organoid foreground segmentation (grey) of Zika infected organoids (14 d.p.i) showing reduction in organoid size, ventricle size, and ventricle count. (**e**) Selected images from Zika-infected organoids showing the compromised morphology of ventricular zones. Images taken from anti-SOX2 staining and inverted. Scale bar = 100 µm. (**f**) Rendering of segmented ventricles in a Zika-infected organoid with their surfaces colored according to detected cytoarchitecture. Scale bar = 200µm. (**g**) Distribution of the mean position of SOX2 cells in virtual cortical columns classified as either "Surface-TBR1" or "Surface-DN" in mock and Zika infected organoids. This measure was used to approximate the average ventricular zone thickness for each group. The overall distribution mean shows a significant drop in mean SOX2 distance from ventricles in Zika infected organoids. (**h**) Correlation of ventricle equivalent diameter with frequency of TBR1 cells detected in virtual cortical columns.

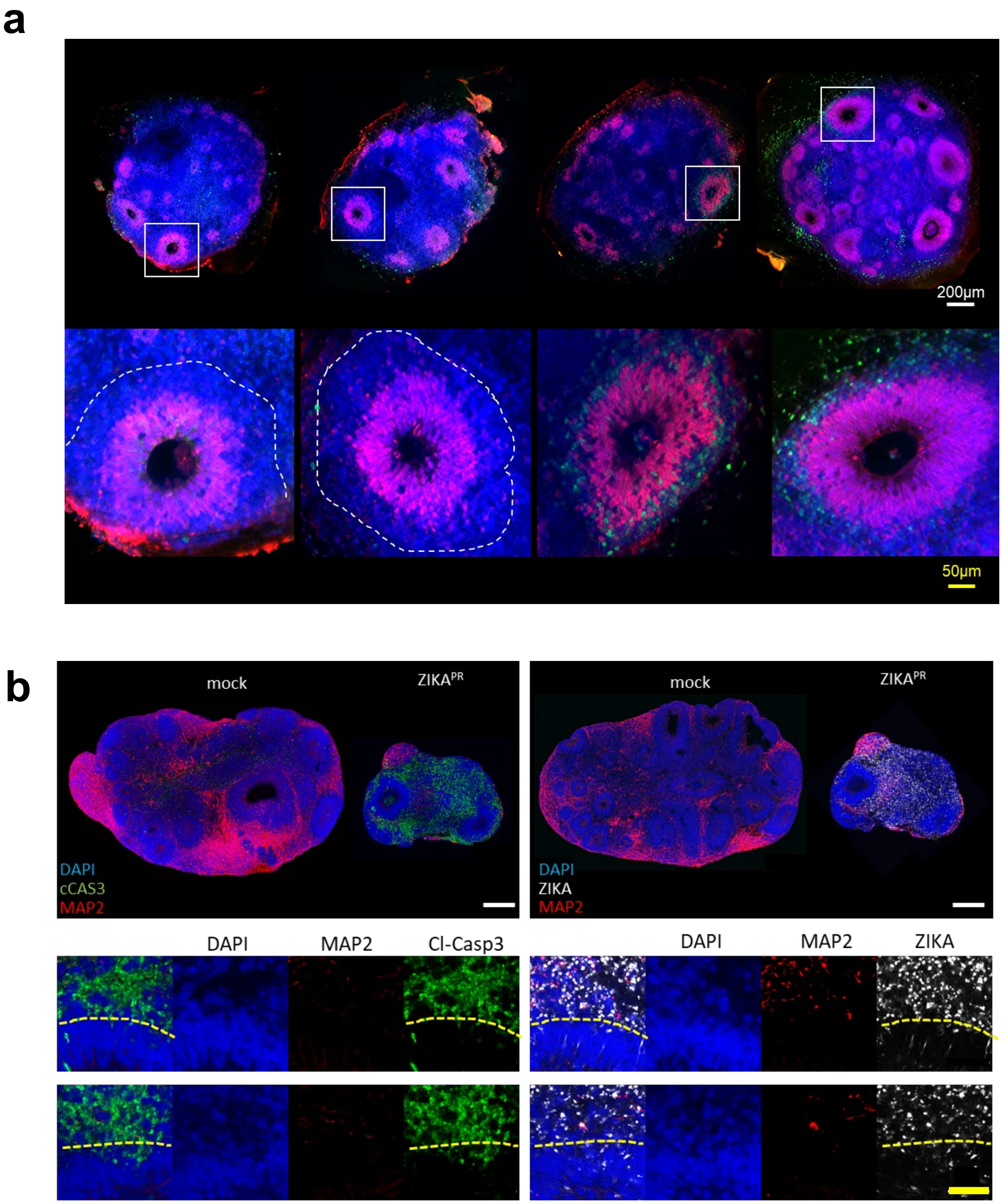

Supplementary Figure 10: Zika DN cells: (**a**) Examples of cell patterns around the ventricles of Zika virus-infected organoids. Pseudo-slices of Zika-infected organoids and close up of square regions. Colors represent Syto16 (blue), SOX2 (red) and TBR1 (green). Two examples on the left show a thick layer of DN cells around the ventricle. Two examples on the right show rare ventricles where a thin layer of TBR1 has formed. White scale bar (top) = 200 µm, yellow scale bar (bottom) = 50 µm. (**b**) Staining of mock and Zika-infected organoids at 14 dpi showing that DN cells surrounding the SOX2+ ventricular zone (dotted line) express Zika envelope protein and are mostly apoptotic. White scale bar (top) = 200 µm, yellow scale bar (bottom) = 50 µm.
