# Supplementary material for: Multiscale 3D phenotyping of human cerebral organoids
Source: Sci Rep. 2020 Dec 8;10:21487. doi: 10.1038/s41598-020-78130-7 (PMC7723053; doi:10.1038/s41598-020-78130-7)
Supplement: Supplementary file 2 — Supplementary Table 1. [file 41598_2020_78130_MOESM2_ESM.pdf]

Supplementary Table 1: List of antibodies used in this study

Antigen	conjugated fluorophore	Catalog #	Vendor	Host	Clonality	Clone
TUBB3	Alexa Fluor 594	657408	Biolegend	ms	M	AA10
MAP2	Alexa Fluor 594	801803	Biolegend	ms	M	SMI 52
TBR1	Alexa Fluor 647	45664S	Cell Signaling Technologies	rb	M	D6C6X
VIMENTIN	Alexa Fluor 647	9856S	Cell Signaling Technologies	rb	M	D21H3
SOX2	none	AF2018	R&D Systems	gt	P	
OTX2	none	AF1979	R&D	gt	P	
REELIN	none	MAB5366	Millipore	ms	M	142
NESTIN	none	MAB5326	Millipore	ms	M	10C2
N-CAD (N-Cadherin)	none	610920	BD Bio	ms	M	32
BLP	none	ABN14	Millipore	rb	P	
FOXG1	none	ab196868	Abcam	rb	M	EPR18987
NKX2.1	none	ab76013	Abcam	rb		
TTR	none	AHP1837	Biorad	sh	P	
PAX6	none	AF8150	R&D	sh	P	
CTIP2	none	ab18465	Abcam	rt	M	25B6
Zika virus Envelope	none	GTX133314	GeneTex	rb	P	
cleaved Caspase-3 (Asp179)	none	9579S	Cell Signaling Technologies	rb	M	D3E9
anti-goat IgG (Fab fragment)	Alexa Fluor 594	805-587-008	Jackson ImmunoResearch	bo	P	
