# Supplementary material for: Multiscale 3D phenotyping of human cerebral organoids
Source: Sci Rep. 2020 Dec 8;10:21487. doi: 10.1038/s41598-020-78130-7 (PMC7723053; doi:10.1038/s41598-020-78130-7)
Supplement: Supplementary file 3 — Supplementary Table 2. [file 41598_2020_78130_MOESM3_ESM.pdf]

Supplementary Table 2: List of the computed features used for inter-scale and comparative analyses

Feature	Scale
DN nbrhd, sox2 count	Single-cell
DN nbrhd, tbr1 count	Single-cell
DN nbrhd, dn count	Single-cell
SOX2 nbrhd, sox2 count	Single-cell
SOX2 nbrhd, tbr1 count	Single-cell
SOX2 nbrhd, dn count	Single-cell
TBR1 nbrhd, sox2 count	Single-cell
TBR1 nbrhd, tbr1 count	Single-cell
TBR1 nbrhd, dn count	Single-cell
DP nbrhd, sox2 count	Single-cell
DP nbrhd, tbr1 count	Single-cell
DP nbrhd, dn count	Single-cell
MidTBR1 nbrhd, sox2 count	Single-cell
MidTBR1 nbrhd, tbr1 count	Single-cell
MidTBR1 nbrhd, dn count	Single-cell
MidSOX2 nbrhd, sox2 count	Single-cell
MidSOX2 nbrhd, tbr1 count	Single-cell
MidSOX2 nbrhd, dn count	Single-cell
MidInter nbrhd, sox2 count	Single-cell
MidInter nbrhd, tbr1 count	Single-cell
MidInter nbrhd, dn count	Single-cell
DN nbrhd fraction	Single-cell
SOX2 nbrhd fraction	Single-cell
TBR1 nbrhd fraction	Single-cell
DP nbrhd fraction	Single-cell
MidTBR1 nbrhd fraction	Single-cell
MidSOX2 nbrhd fraction	Single-cell
MidInter nbrhd fraction	Single-cell
TBR1 / SOX2 ratio	Single-cell
MidTBR1 / MidSOX2 ratio	Single-cell
MidInter / DN ratio	Single-cell
DN nbrhd, eq diam mean	Single-cell
DN nbrhd, eq diam stdev	Single-cell
SOX2 nbrhd, eq diam mean	Single-cell
SOX2 nbrhd, eq diam stdev	Single-cell
TBR1 nbrhd, eq diam mean	Single-cell
TBR1 nbrhd, eq diam stdev	Single-cell
DP nbrhd, eq diam mean	Single-cell
DP nbrhd, eq diam stdev	Single-cell
MidTBR1 nbrhd, eq diam mean	Single-cell
MidTBR1 nbrhd, eq diam stdev	Single-cell
MidSOX2 nbrhd, eq diam mean	Single-cell
MidSOX2 nbrhd, eq diam stdev	Single-cell
MidInter nbrhd, eq diam mean	Single-cell
MidInter nbrhd, eq diam stdev	Single-cell
DN nbrhd, major axis mean	Single-cell
DN nbrhd, major axis stdev	Single-cell
SOX2 nbrhd, major axis mean	Single-cell
SOX2 nbrhd, major axis stdev	Single-cell
TBR1 nbrhd, major axis mean	Single-cell
TBR1 nbrhd, major axis stdev	Single-cell
DP nbrhd, major axis mean	Single-cell
DP nbrhd, major axis stdev	Single-cell
MidTBR1 nbrhd, major axis mean	Single-cell
MidTBR1 nbrhd, major axis stdev	Single-cell
MidSOX2 nbrhd, major axis mean	Single-cell
MidSOX2 nbrhd, major axis stdev	Single-cell
MidInter nbrhd, major axis mean	Single-cell
MidInter nbrhd, major axis stdev	Single-cell
DN nbrhd, axis ratio mean	Single-cell
DN nbrhd, axis ratio stdev	Single-cell
SOX2 nbrhd, axis ratio mean	Single-cell
SOX2 nbrhd, axis ratio stdev	Single-cell

TBR1 nbrhd, axis ratio mean	Single-cell
TBR1 nbrhd, axis ratio stdev	Single-cell
DP nbrhd, axis ratio mean	Single-cell
DP nbrhd, axis ratio stdev	Single-cell
MidTBR1 nbrhd, axis ratio mean	Single-cell
MidTBR1 nbrhd, axis ratio stdev	Single-cell
MidSOX2 nbrhd, axis ratio mean	Single-cell
MidSOX2 nbrhd, axis ratio stdev	Single-cell
MidInter nbrhd, axis ratio mean	Single-cell
MidInter nbrhd, axis ratio stdev	Single-cell
DN nbrhd, sox2 proximity to SOX2 mean	Single-cell
DN nbrhd, sox2 proximity to SOX2 stdev	Single-cell
DN nbrhd, sox2 proximity to TBR1 mean	Single-cell
DN nbrhd, sox2 proximity to TBR1 stdev	Single-cell
DN nbrhd, tbr1 proximity to SOX2 mean	Single-cell
DN nbrhd, tbr1 proximity to SOX2 stdev	Single-cell
DN nbrhd, tbr1 proximity to TBR1 mean	Single-cell
DN nbrhd, tbr1 proximity to TBR1 stdev	Single-cell
DN nbrhd, dn proximity to SOX2 mean	Single-cell
DN nbrhd, dn proximity to SOX2 stdev	Single-cell
DN nbrhd, dn proximity to TBR1 mean	Single-cell
DN nbrhd, dn proximity to TBR1 stdev	Single-cell
SOX2 nbrhd, sox2 proximity to SOX2 mean	Single-cell
SOX2 nbrhd, sox2 proximity to SOX2 stdev	Single-cell
SOX2 nbrhd, sox2 proximity to TBR1 mean	Single-cell
SOX2 nbrhd, sox2 proximity to TBR1 stdev	Single-cell
SOX2 nbrhd, tbr1 proximity to SOX2 mean	Single-cell
SOX2 nbrhd, tbr1 proximity to SOX2 stdev	Single-cell
SOX2 nbrhd, tbr1 proximity to TBR1 mean	Single-cell
SOX2 nbrhd, tbr1 proximity to TBR1 stdev	Single-cell
SOX2 nbrhd, dn proximity to SOX2 mean	Single-cell
SOX2 nbrhd, dn proximity to SOX2 stdev	Single-cell
SOX2 nbrhd, dn proximity to TBR1 mean	Single-cell
SOX2 nbrhd, dn proximity to TBR1 stdev	Single-cell
TBR1 nbrhd, sox2 proximity to SOX2 mean	Single-cell
TBR1 nbrhd, sox2 proximity to SOX2 stdev	Single-cell
TBR1 nbrhd, sox2 proximity to TBR1 mean	Single-cell
TBR1 nbrhd, sox2 proximity to TBR1 stdev	Single-cell
TBR1 nbrhd, tbr1 proximity to SOX2 mean	Single-cell
TBR1 nbrhd, tbr1 proximity to SOX2 stdev	Single-cell
TBR1 nbrhd, tbr1 proximity to TBR1 mean	Single-cell
TBR1 nbrhd, tbr1 proximity to TBR1 stdev	Single-cell
TBR1 nbrhd, dn proximity to SOX2 mean	Single-cell
TBR1 nbrhd, dn proximity to SOX2 stdev	Single-cell
TBR1 nbrhd, dn proximity to TBR1 mean	Single-cell
TBR1 nbrhd, dn proximity to TBR1 stdev	Single-cell
DP nbrhd, sox2 proximity to SOX2 mean	Single-cell
DP nbrhd, sox2 proximity to SOX2 stdev	Single-cell
DP nbrhd, sox2 proximity to TBR1 mean	Single-cell
DP nbrhd, sox2 proximity to TBR1 stdev	Single-cell
DP nbrhd, tbr1 proximity to SOX2 mean	Single-cell
DP nbrhd, tbr1 proximity to SOX2 stdev	Single-cell
DP nbrhd, tbr1 proximity to TBR1 mean	Single-cell
DP nbrhd, tbr1 proximity to TBR1 stdev	Single-cell
DP nbrhd, dn proximity to SOX2 mean	Single-cell
DP nbrhd, dn proximity to SOX2 stdev	Single-cell
DP nbrhd, dn proximity to TBR1 mean	Single-cell
DP nbrhd, dn proximity to TBR1 stdev	Single-cell
MidTBR1 nbrhd, sox2 proximity to SOX2 mean	Single-cell
MidTBR1 nbrhd, sox2 proximity to SOX2 stdev	Single-cell
MidTBR1 nbrhd, sox2 proximity to TBR1 mean	Single-cell
MidTBR1 nbrhd, sox2 proximity to TBR1 stdev	Single-cell
MidTBR1 nbrhd, tbr1 proximity to SOX2 mean	Single-cell
MidTBR1 nbrhd, tbr1 proximity to SOX2 stdev	Single-cell
MidTBR1 nbrhd, tbr1 proximity to TBR1 mean	Single-cell

MidTBR1 nbrhd, tbr1 proximity to TBR1 stdev	Single-cell
MidTBR1 nbrhd, dn proximity to SOX2 mean	Single-cell
MidTBR1 nbrhd, dn proximity to SOX2 stdev	Single-cell
MidTBR1 nbrhd, dn proximity to TBR1 mean	Single-cell
MidTBR1 nbrhd, dn proximity to TBR1 stdev	Single-cell
MidSOX2 nbrhd, sox2 proximity to SOX2 mean	Single-cell
MidSOX2 nbrhd, sox2 proximity to SOX2 stdev	Single-cell
MidSOX2 nbrhd, sox2 proximity to TBR1 mean	Single-cell
MidSOX2 nbrhd, sox2 proximity to TBR1 stdev	Single-cell
MidSOX2 nbrhd, tbr1 proximity to SOX2 mean	Single-cell
MidSOX2 nbrhd, tbr1 proximity to SOX2 stdev	Single-cell
MidSOX2 nbrhd, tbr1 proximity to TBR1 mean	Single-cell
MidSOX2 nbrhd, tbr1 proximity to TBR1 stdev	Single-cell
MidSOX2 nbrhd, dn proximity to SOX2 mean	Single-cell
MidSOX2 nbrhd, dn proximity to SOX2 stdev	Single-cell
MidSOX2 nbrhd, dn proximity to TBR1 mean	Single-cell
MidSOX2 nbrhd, dn proximity to TBR1 stdev	Single-cell
MidInter nbrhd, sox2 proximity to SOX2 mean	Single-cell
MidInter nbrhd, sox2 proximity to SOX2 stdev	Single-cell
MidInter nbrhd, sox2 proximity to TBR1 mean	Single-cell
MidInter nbrhd, sox2 proximity to TBR1 stdev	Single-cell
MidInter nbrhd, tbr1 proximity to SOX2 mean	Single-cell
MidInter nbrhd, tbr1 proximity to SOX2 stdev	Single-cell
MidInter nbrhd, tbr1 proximity to TBR1 mean	Single-cell
MidInter nbrhd, tbr1 proximity to TBR1 stdev	Single-cell
MidInter nbrhd, dn proximity to SOX2 mean	Single-cell
MidInter nbrhd, dn proximity to SOX2 stdev	Single-cell
MidInter nbrhd, dn proximity to TBR1 mean	Single-cell
MidInter nbrhd, dn proximity to TBR1 stdev	Single-cell
TBR1-LowDN Cytoarchitecture fraction	Cytoarchitecture
TBR1-HighDN Cytoarchitecture fraction	Cytoarchitecture
Surface Cytoarchitecture fraction	Cytoarchitecture
Artifacts Cytoarchitecture fraction	Cytoarchitecture
DN Cytoarchitecture fraction	Cytoarchitecture
Adjacent Cytoarchitecture fraction	Cytoarchitecture
ave. TBR1-LowDN profile, sox2 count	Cytoarchitecture
ave. TBR1-LowDN profile, tbr1 count	Cytoarchitecture
ave. TBR1-LowDN profile, dn count	Cytoarchitecture
ave. TBR1-HighDN profile, sox2 count	Cytoarchitecture
ave. TBR1-HighDN profile, tbr1 count	Cytoarchitecture
ave. TBR1-HighDN profile, dn count	Cytoarchitecture
ave. Surface profile, sox2 count	Cytoarchitecture
ave. Surface profile, tbr1 count	Cytoarchitecture
ave. Surface profile, dn count	Cytoarchitecture
ave. Artifacts profile, sox2 count	Cytoarchitecture
ave. Artifacts profile, tbr1 count	Cytoarchitecture
ave. Artifacts profile, dn count	Cytoarchitecture
ave. DN profile, sox2 count	Cytoarchitecture
ave. DN profile, tbr1 count	Cytoarchitecture
ave. DN profile, dn count	Cytoarchitecture
ave. Adjacent profile, sox2 count	Cytoarchitecture
ave. Adjacent profile, tbr1 count	Cytoarchitecture
ave. Adjacent profile, dn count	Cytoarchitecture
ave. TBR1-LowDN profile, sox2 mean position	Cytoarchitecture
ave. TBR1-LowDN profile, tbr1 mean position	Cytoarchitecture
ave. TBR1-LowDN profile, dn mean position	Cytoarchitecture
ave. TBR1-HighDN profile, sox2 mean position	Cytoarchitecture
ave. TBR1-HighDN profile, tbr1 mean position	Cytoarchitecture
ave. TBR1-HighDN profile, dn mean position	Cytoarchitecture
ave. Surface profile, sox2 mean position	Cytoarchitecture
ave. Surface profile, tbr1 mean position	Cytoarchitecture
ave. Surface profile, dn mean position	Cytoarchitecture
ave. Artifacts profile, sox2 mean position	Cytoarchitecture
ave. Artifacts profile, tbr1 mean position	Cytoarchitecture
ave. Artifacts profile, dn mean position	Cytoarchitecture

ave. DN profile, sox2 mean position	Cytoarchitecture
ave. DN profile, tbr1 mean position	Cytoarchitecture
ave. DN profile, dn mean position	Cytoarchitecture
ave. Adjacent profile, sox2 mean position	Cytoarchitecture
ave. Adjacent profile, tbr1 mean position	Cytoarchitecture
ave. Adjacent profile, dn mean position	Cytoarchitecture
ave. TBR1-LowDN profile, sox2 profile stdev	Cytoarchitecture
ave. TBR1-LowDN profile, tbr1 profile stdev	Cytoarchitecture
ave. TBR1-LowDN profile, dn profile stdev	Cytoarchitecture
ave. TBR1-HighDN profile, sox2 profile stdev	Cytoarchitecture
ave. TBR1-HighDN profile, tbr1 profile stdev	Cytoarchitecture
ave. TBR1-HighDN profile, dn profile stdev	Cytoarchitecture
ave. Surface profile, sox2 profile stdev	Cytoarchitecture
ave. Surface profile, tbr1 profile stdev	Cytoarchitecture
ave. Surface profile, dn profile stdev	Cytoarchitecture
ave. Artifacts profile, sox2 profile stdev	Cytoarchitecture
ave. Artifacts profile, tbr1 profile stdev	Cytoarchitecture
ave. Artifacts profile, dn profile stdev	Cytoarchitecture
ave. DN profile, sox2 profile stdev	Cytoarchitecture
ave. DN profile, tbr1 profile stdev	Cytoarchitecture
ave. DN profile, dn profile stdev	Cytoarchitecture
ave. Adjacent profile, sox2 profile stdev	Cytoarchitecture
ave. Adjacent profile, tbr1 profile stdev	Cytoarchitecture
ave. Adjacent profile, dn profile stdev	Cytoarchitecture
organoid volume (mm3)	Whole-organoid
organoid equivalent diameter (mm)	Whole-organoid
organoid major axis (mm)	Whole-organoid
organoid minor axis (mm)	Whole-organoid
organoid axis ratio	Whole-organoid
ventricle count	Whole-organoid
ventricle volume mean (um3)	Whole-organoid
ventricle volume stdev (um3)	Whole-organoid
ventricle equivalent diameter mean (um)	Whole-organoid
ventricle equivalent diameter stdev (um)	Whole-organoid
ventricle major axis mean (um)	Whole-organoid
ventricle major axis stdev (um)	Whole-organoid
ventricle minor axis mean (um)	Whole-organoid
ventricle minor axis stdev (um)	Whole-organoid
ventricle axis ratio mean	Whole-organoid
ventricle axis ratio stdev	Whole-organoid
DN nbrhd, sox2 surface distance mean (um)	Whole-organoid
DN nbrhd, sox2 surface distance stdev (um)	Whole-organoid
DN nbrhd, tbr1 surface distance mean (um)	Whole-organoid
DN nbrhd, tbr1 surface distance stdev (um)	Whole-organoid
DN nbrhd, dn surface distance mean (um)	Whole-organoid
DN nbrhd, dn surface distance stdev (um)	Whole-organoid
SOX2 nbrhd, sox2 surface distance mean (um)	Whole-organoid
SOX2 nbrhd, sox2 surface distance stdev (um)	Whole-organoid
SOX2 nbrhd, tbr1 surface distance mean (um)	Whole-organoid
SOX2 nbrhd, tbr1 surface distance stdev (um)	Whole-organoid
SOX2 nbrhd, dn surface distance mean (um)	Whole-organoid
SOX2 nbrhd, dn surface distance stdev (um)	Whole-organoid
TBR1 nbrhd, sox2 surface distance mean (um)	Whole-organoid
TBR1 nbrhd, sox2 surface distance stdev (um)	Whole-organoid
TBR1 nbrhd, tbr1 surface distance mean (um)	Whole-organoid
TBR1 nbrhd, tbr1 surface distance stdev (um)	Whole-organoid
TBR1 nbrhd, dn surface distance mean (um)	Whole-organoid
TBR1 nbrhd, dn surface distance stdev (um)	Whole-organoid
DP nbrhd, sox2 surface distance mean (um)	Whole-organoid
DP nbrhd, sox2 surface distance stdev (um)	Whole-organoid
DP nbrhd, tbr1 surface distance mean (um)	Whole-organoid
DP nbrhd, tbr1 surface distance stdev (um)	Whole-organoid
DP nbrhd, dn surface distance mean (um)	Whole-organoid
DP nbrhd, dn surface distance stdev (um)	Whole-organoid
MidTBR1 nbrhd, sox2 surface distance mean (um)	Whole-organoid

MidTBR1 nbrhd, sox2 surface distance stdev (um)	Whole-organoid
MidTBR1 nbrhd, tbr1 surface distance mean (um)	Whole-organoid
MidTBR1 nbrhd, tbr1 surface distance stdev (um)	Whole-organoid
MidTBR1 nbrhd, dn surface distance mean (um)	Whole-organoid
MidTBR1 nbrhd, dn surface distance stdev (um)	Whole-organoid
MidSOX2 nbrhd, sox2 surface distance mean (um)	Whole-organoid
MidSOX2 nbrhd, sox2 surface distance stdev (um)	Whole-organoid
MidSOX2 nbrhd, tbr1 surface distance mean (um)	Whole-organoid
MidSOX2 nbrhd, tbr1 surface distance stdev (um)	Whole-organoid
MidSOX2 nbrhd, dn surface distance mean (um)	Whole-organoid
MidSOX2 nbrhd, dn surface distance stdev (um)	Whole-organoid
MidInter nbrhd, sox2 surface distance mean (um)	Whole-organoid
MidInter nbrhd, tbr1 surface distance mean (um)	Whole-organoid
MidInter nbrhd, tbr1 surface distance stdev (um)	Whole-organoid
MidInter nbrhd, dn surface distance mean (um)	Whole-organoid
MidInter nbrhd, dn surface distance stdev (um)	Whole-organoid
MidInter nbrhd, dn surface distance stdev (um)	Whole-organoid
