# Supplementary material for: Multiscale 3D phenotyping of human cerebral organoids
Source: Sci Rep. 2020 Dec 8;10:21487. doi: 10.1038/s41598-020-78130-7 (PMC7723053; doi:10.1038/s41598-020-78130-7)
Supplement: Supplementary file 4 — Supplementary Information. [file 41598_2020_78130_MOESM4_ESM.pdf]

Walkthrough with test data

The following walkthrough illustrates how to use SCOUT on a small organoid dataset using Docker. Docker provides a cross-platform way to run SCOUT with minimal setup. The test dataset contains stitched (whole-organoid) syto16, SOX2, and TBR1 images from a portion of a d35 cerebral organoid cultured similar to the Lancaster protocol (described in the Methods section).

Before you start

This walkthrough has been optimized on Ubuntu Linux, so other platforms may require slight changes to the commands below. Windows and Mac users may need to make the following adjustments to the walkthrough commands:

1. **Remove *sudo* from all commands**
    - If Docker is available to non-root users, then sudo is not needed on Mac. On Windows, *sudo* is not available.
2. **Replace *$(pwd)* with a full path to *scout-data/test***
    - This may work on Mac, but using this subshell syntax on Windows Powershell would not work. This should be replaced with `C:\path\to\scout-data\test`.
3. **Remove the line breaks**
    - The backslash syntax for line breaks used in this walkthrough may not work on Windows Powershell. Instead, you can enter the command using a single line.
4. **Make sure Docker has permission to mount the drive with *scout-data/***
    - On Windows, Docker may only have permissions to access the *C:* drive by default. If *scout-data* is placed on another drive, then you should give Docker proper permissions by accessing the Docker settings through the Docker tray icon.

Docker setup

The easiest way to ensure a similar runtime environment for SCOUT on Windows, Mac, and Linux is by using the `chunglabmit/scout` Docker image hosted on `Dockerhub`. First, you will need to install “Docker Desktop” (which is free) by following the platform-specific instructions at https://docs.docker.com/get-docker/

Once installed, you will need to download the pre-built Docker image for SCOUT. Open a terminal and use `docker pull` to download it:

```
sudo docker pull chunglabmit/scout
```

This image may be moved to `chunglabmit/scout` in the future. Note that the *sudo* keyword may not be needed on your platform for Docker commands.

Docker Desktop on Windows and Mac may restrict the amount of CPU and RAM resources that each container can use by default. You can adjust resource allocation by accessing the Docker settings through the Docker tray icon. You may also need to allow Docker access to other drives (D:, E:, etc) if the `scout-data` directory is placed on a different drive. Lastly, if you want to run Jupyter notebooks with the SCOUT Docker image, you may need to follow some platform-specific networking setup (port forwarding, routing, etc), which you can read more about at https://docs.docker.com/docker-for-windows/networking/ or https://docs.docker.com/docker-for-mac/networking/ for Windows and Mac, respectively.

Download test data

After installing the SCOUT Docker image, a small test dataset (~3 GB) can be downloaded from Dropbox. The test dataset is distributed as an archive called `scout-data.zip`, which contains two subfolders: `test` and `results`. The `test` folder contains all the data needed to start the SCOUT analysis from the begining, such as raw stitched images from a microscope. The `results` folder contains all the intermediate results expected from completing the following walkthrough. This data is included for completeness and verification purposes and is not required to actually run SCOUT on newly acquired data.

First, download the test dataset from https://www.dropbox.com/s/j37p5m7q7qk1mp1/scout-data.zip?dl=0 and unzip it. Make note of the resulting `scout-data/test` and `scout-data/results` folders.

Open a terminal (or Powershell on Windows) and move into the `scout-data/test` directory:

```
cd path/to/scout-data/test  # Replace with actual path
```

This folder will be mounted into the SCOUT Docker container using the `-v ...:/scout/data` argument to `docker run` throughout the following walkthrough.

Preprocessing

The first step in the SCOUT pipeline is to estimate the overall image histograms for each channel.

```
sudo docker run -v "$(pwd):/scout/data" chunglabmit/scout preprocess histogram \
    data/dataset/color_0/ data/dataset/color0_hist.csv -s 1 -v
sudo docker run -v "$(pwd):/scout/data" chunglabmit/scout preprocess histogram \
    data/dataset/color_1/ data/dataset/color1_hist.csv -s 1 -v
sudo docker run -v "$(pwd):/scout/data" chunglabmit/scout preprocess histogram \
    data/dataset/color_2/ data/dataset/color2_hist.csv -s 1 -v
```

This will create 3 CSV files with histograms for each channel. Using these histograms, we can normalize the images to the range [0, 1] and apply a background threshold.

```
sudo docker run -v "$(pwd):/scout/data" chunglabmit/scout preprocess rescale \
    data/dataset/color_0/ data/dataset/color0_hist.csv data/dataset/color0_rescaled \
    -t 120 -p 99.7 -v
sudo docker run -v "$(pwd):/scout/data" chunglabmit/scout preprocess rescale \
    data/dataset/color_1/ data/dataset/color1_hist.csv data/dataset/color1_rescaled \
    -t 100 -p 99.7 -v
sudo docker run -v "$(pwd):/scout/data" chunglabmit/scout preprocess rescale \
    data/dataset/color_2/ data/dataset/color2_hist.csv data/dataset/color2_rescaled \
    -t 100 -p 99.7 -v
```

This will create three new folders containing normalized TIFF images for each channel. In order to more easily work with volumetric image data, we the convert the 2D TIFF stacks into 3D Zarr arrays. Each Zarr array is a nested folder of chunk compressed voxel data. By default, the chunk size is (64, 64, 64).

```
sudo docker run -v "$(pwd):/scout/data" chunglabmit/scout preprocess convert \
    data/dataset/color0_rescaled data/dataset/syto.zarr -v
sudo docker run -v "$(pwd):/scout/data" chunglabmit/scout preprocess convert \
    data/dataset/color1_rescaled data/dataset/sox2.zarr -v
sudo docker run -v "$(pwd):/scout/data" chunglabmit/scout preprocess convert \
    data/dataset/color2_rescaled data/dataset/tbr1.zarr -v
```

This will create three new `*.zarr` folders, one for each channel.

Nuclei Detection

Once we have the syto16.zarr array, we can detect nuclei centroids using parallel processing on each image chunk. Note that the current Docker image does not support GPU acceleration, and this step would be much faster by installing from source on a machine with a GPU.

```
sudo docker run -v "$(pwd):/scout/data" chunglabmit/scout nuclei detect data/dataset/syto.zarr \
    data/dataset/nuclei_probability.zarr data/dataset/centroids.npy \
    --voxel-size data/dataset/voxel_size.csv \
    --output-um data/dataset/centroids_um.npy -n 2 -v
```

This will create a new Zarr array, `nuclei_probability.zarr`, as well as two numpy arrays with nuclei centroid coordinates. Given these nuclei centroids, we can perform a seeded watershed segmentation of the nuclei probability array to obtain the shape of each detected nucleus. This operation is done with some overlap between adjacent chunks to avoid artifacts at the boundaries between adjacent chunks in the watershed lines.

```
sudo docker run -v "$(pwd):/scout/data" chunglabmit/scout nuclei segment \
    data/dataset/nuclei_probability.zarr data/dataset/centroids.npy \
    data/dataset/nuclei_foreground.zarr data/dataset/nuclei_binary.zarr -n 2 -v
```

This will create two new Zarr arrays, `nuclei_foreground.zarr` and `nuclei_binary.zarr`. Given this binary nuclei segmentation, we can compute morphological features for each nucleus. The resulting morphological features are stored in a CSV.

```
sudo docker run -v "$(pwd):/scout/data" chunglabmit/scout nuclei morphology \
    data/dataset/nuclei_binary.zarr data/dataset/centroids.npy \
    data/dataset/nuclei_morphologies.csv -v
```

This will create a CSV file containing multiple morphological measurements for each segmented nucleus. Finally, we can sample the fluorescence in the other channels (SOX2 and TBR1 in this case) at each nucleus centroid.

```
sudo docker run -v "$(pwd):/scout/data" chunglabmit/scout nuclei fluorescence \
    data/dataset/centroids.npy data/dataset/nuclei_fluorescence/ \
    data/dataset/sox2.zarr/ data/dataset/tbr1.zarr/ -v
```

This will create a folder, `nuclei_fluorescence/`, that contains numpy arrays with the fluorescence mean and standard deviation for each detected nucleus. The resulting mean fluorescence intensities (MFIs) are useful for gating cells into different cell types based on protein expression.

```
sudo docker run -v "$(pwd):/scout/data" chunglabmit/scout nuclei gate \
    data/dataset/nuclei_fluorescence/nuclei_mfis.npy \
    data/dataset/nuclei_gating.npy 0.35 0.25 -v
```

This will create a numpy array, `nuclei_gating.npy`, containing binary cell type labels for each

nucleus. In this case, high SOX2 expression is used to identify neural progenitors and high TBR1 expression is used to identify post-motitic neurons. Cells that have low SOX2 and TBR1 expression are called “double negative” (DN). Cell types can be named in order using the following command:

```
sudo docker run -v "$(pwd):/scout/data" chunglabmit/scout nuclei name \
    sox2 tbr1 dn -o data/dataset/celltype_names.csv -v
```

This will create a CSV file with names for each cell type.

Microenvironment Analysis

(Note that this was formerly called *niche* analysis)

Given nuclei centroids and cell type labels, we can further describe the microenvironment around each cell. To do this, we compute the *proximity* to each of the non-DN cell types, which is described in the Method section.

```
sudo docker run -v "$(pwd):/scout/data" chunglabmit/scout niche proximity \
    data/dataset/centroids_um.npy data/dataset/nuclei_gating.npy \
    data/dataset/niche_proximities.npy -r 25 25 -k 2 -v
```

This will create a numpy array with proximities to each cell type. These spatial proximities are attibutes of each cell describing the local environment. The next step is to use these proximity values to further gate cells into subpopulations based on their spatial context.

```
sudo docker run -v "$(pwd):/scout/data" chunglabmit/scout niche gate \
    data/dataset/niche_proximities.npy data/dataset/niche_labels.npy \
    --low 0.2 0.2 --high 0.66 0.63 -v
```

This will create a numpy array containing microenvironment labels for each nucleus. Here, we defined a *low* and high` proximity threshold for SOX2 and TBR1 separately. This results in 7 subpopulations (3 high, 3 mid, and 1 low), which can be named using the following command:

```
sudo docker run -v "$(pwd):/scout/data" chunglabmit/scout niche name \
    DN SOX2 TBR1 DP MidTBR1 MidSOX2 MidInter -o data/dataset/niche_names.csv -v
```

This will create a CSV file with names for each microenvironment.

Ventricle Segmentation

Next, we turn to ventricle segmentation, which is required to calculate radial profiles in a cytoarchitecture analysis. The pretrained U-Net model assumes that each input image is of nuclear staining at 4 um pixel resoltion. We, therefore, resize the normalized nuclei images and stack them into a single 3D TIFF.

```
sudo docker run -v "$(pwd):/scout/data" chunglabmit/scout segment downsample \
    data/dataset/color0_rescaled/ data/dataset/syto_down6x 6 6 -v -t
sudo docker run -v "$(pwd):/scout/data" chunglabmit/scout segment stack \
    data/dataset/syto_down6x/ data/dataset/syto_down6x.tif -v
```

This will create a new folder and 3D TIFF with 6x downsampled (in x and y) images. The 3D TIFF can be passed to the U-Net model for ventricle segmentation, which occurs one 2D slide at a time.

```
sudo docker run -v "$(pwd):/scout/data" chunglabmit/scout segment ventricle \
    data/dataset/syto_down6x.tif models/unet_weights3_zika.h5 \
    data/dataset/segment_ventricles.tif -t 0.5 -v
```

This will result in a 3D TIFF, `segment_ventricles.tif`, containing a binary segmentation of all ventricles. We also need a foreground segmentation to determine the overall organoid size and shape. A foreground segmentation can be computed by thresholding.

```
sudo docker run -v "$(pwd):/scout/data" chunglabmit/scout segment foreground \
    data/dataset/syto_down6x.tif data/dataset/segment_foreground.tif -v -t 0.02 -g 8 4 4
```

This will create another 3D TIFF, `segment_foreground.tif`, containing a binary segmentation of the whole organoid.

Cytoarchitecture Analysis

Given the ventricle segmentation, nuclei centroids, and cell types labels, radial profiles from each ventricle can be computed. First, the ventricle segmentation is turned into a polygon mesh (using the marching cubes algorithm).

```
sudo docker run -v "$(pwd):/scout/data" chunglabmit/scout cyto mesh \
    data/dataset/segment_ventricles.tif data/dataset/voxel_size.csv \
    data/dataset/mesh_ventricles.pkl -d 1 6 6 -g 2 -s 3 -v
```

This will generate a pickled Python dictionary, `mesh_ventricles.pkl`, containing mesh verticies, faces, and normals. Then, normal vectors from this mesh are used to compute radial profiles for each cell type.

```
sudo docker run -v "$(pwd):/scout/data" chunglabmit/scout cyto profiles \
    data/dataset/mesh_ventricles.pkl data/dataset/centroids_um.npy \
    data/dataset/nuclei_gating.npy data/dataset/cyto_profiles.npy -v
```

This will create a numpy array, `cyto_profiles.npy`, containing radial profiles of cell counts. Finally, we randomly sample from the large number of radial profiles to be able to cluster radial profiles across many organoids. This step isn't required in this case, but we include it for completeness.

```
sudo docker run -v "$(pwd):/scout/data" chunglabmit/scout cyto sample 5000 \
    data/dataset/cyto_sample_index.npy -i data/dataset/cyto_profiles.npy \
    -o data/dataset/cyto_profiles_sample.npy -v
```

This will create numpy arrays containing a random sample of radial profiles and the corresponding index from the original array of profiles. Then, we would compute clusters of cytoarchitectures across all organoids by combining sampled profiles and using the `determine cyto clusters.ipynb` notebook. You can access and use these notebooks by starting a Jupyter server within the SCOUT Docker container:

```
sudo docker run -it -v "$(pwd):/scout/data" -p 8888:8888 chunglabmit/scout jupyter --ip 0.0.0.0
```

Note that the positions of the *-p* and *–ip* arguments are important because *-p* is for Docker port forwarding and *–ip* is for the Jupyter server. You can navigate to `localhost:8888` in your browser and copy the access token printed to the terminal as `/?token={copy-this-text}`.

For the sake of brevity, we simply provide precomputed profiles, labels, and a fit UMAP model from our d35/d60 comparison. With these, we can classify the cytoarchitecture of all radial profiles.

```
sudo docker run -v "$(pwd):/scout/data" chunglabmit/scout cyto classify \
    data/cyto_profiles_combined.npy data/cyto_labels_combined.npy \
    data/dataset/cyto_profiles.npy data/cyto_labels.npy -v \
    --umap data/model_d35_d60.umap
```

This will create a numpy array, `cyto_labels.npy`, containing cytoarchitecture labels for each radial profile. Note that because the test dataset is not a full 3D dataset, the resulting radial profiles and cytoarchitecture labels obtained here may have some artifacts due to empty profiles near the top and bottom of the test volume.

We can provide appropriate names for each cytoarchitecture cluster after inspecting each cluster in the `determine cyto clusters.ipynb` notebook.

```
sudo docker run -v "$(pwd):/scout/data" chunglabmit/scout cyto name \
TBR1-LowDN TBR1-HighDN Surface Artifacts DN Adjacent -o data/cyto_names.csv -v
```

This will create a CSV with names for each cytoarchitecture class.

Multiscale Analysis

All of the intermediate results above are used to compute multiscale features for each dataset in an analysis. Note that the following command assumes that the intermediate results are named as shown in the previous steps.

```
sudo docker run -v "$(pwd):/scout/data" chunglabmit/scout multiscale features data/ \
    -d 1 6 6 -g 2 -v
```

This command will create an Excel file called `organoid_features.xlsx`, which is the final step in the walkthrough. Details of how to perform statistical analysis of multiple organoids can be found in the full SCOUT tutorial.

Expected results

The final `organoid_features.xlsx` file can be inspected in Excel. For convenience, we highlight some expected results in `organoid_features.xlsx` here:

- TBR1 nbrhd, tbr count: 3967
- SOX2 nbrhd, sox2 count: 12670
- ventricle equivalent diameter mean (um): 48.874
- organoid volume (mm3): 0.06565 *(not a full organoid dataset)*

All of the intermediate results can be compared to the results in `scout-data/results`.
