# Supplementary material for: Multiscale 3D phenotyping of human cerebral organoids
Source: Sci Rep. 2020 Dec 8;10:21487. doi: 10.1038/s41598-020-78130-7 (PMC7723053; doi:10.1038/s41598-020-78130-7)
Supplement: Supplementary file 5 — Supplementary Video Legends. [file 41598_2020_78130_MOESM5_ESM.docx]

**Supplemental Video Legends**:

**Video S1: Day 35 organoid.** Video demonstrating flythrough and 3D render of a day 35 cerebral organoid. Organoids were SHIELD processed, delipidated, antibody stained with eFLASH and imaged using LSFM. Video shows Syto16-labeled nuclei (blue), SOX2 (red) and TBR1 (green).

**Video S2: Day 60 organoid.** Video demonstrating flythrough and 3D render of a day 60 cerebral organoid labeled with Syto16 (blue), anti-SOX2 (red) and anti-TBR1 (green).

**Video S3:** Video demonstrating flythrough and 3D render of a Velasco day 56 cerebral organoid labeled with Syto16 (blue), anti-SOX2 (red) and anti-TBR1 (green).

**Video S4: Velasco d56 organoid. Zika-infected organoid.** Video demonstrating flythrough of Zika virus (Puerto Rico isolate) infected organoid 14 days post infection labeled with Syto16 (blue), anti-SOX2 (red) and anti-TBR1 (green).
